# Deep learning-based genetic disease prediction with Monte Carlo (MC) dropout uncertainty estimation: a comparative study with classical machine learning baselines

**DOI:** 10.3389/fgene.2026.1969295

**Published:** 2026-10-19

**Authors:** V. S. Keerthana, Tarak Hussain

**Affiliations:** Department of Computer Science & Engineering, Koneru Lakshmaiah Education Foundation, Vaddeswaram, India

**Keywords:** Bayesian deep learning, clinical decision support, deep learning, genetic disease prediction, machine learning benchmarking, model calibration, Monte Carlo (MC) dropout, precision medicine

## Abstract

Background: Deep neural networks have been successfully applied to structured clinical data with a good predictive performance, but their point predictions do not reflect an indication of confidence, which limits their trustworthiness in screening for genetic conditions where an overconfident misclassification may delay the appropriate confirmatory testing or intervention. Methods: A feed-forward neural network (128-64-32 hidden units, batch normalization, and dropout p = 0.3) was trained to classify patients into five genetic-disease-associated groups based on twelve regularly accessible clinical and molecular biomarkers, using a publicly available, pre-labelled structured biomedical dataset (n = 1,000) whose real-world clinical vs. synthetic provenance could not be independently confirmed (see Section 2.1 and Limitations). Performance was assessed using stratified 5-fold cross-validation versus four traditional baselines (logistic regression, random forest, SVM, gradient boosting; McNemar’s test for pairwise significance). We quantified predictive uncertainty using Monte Carlo (MC) Dropout (T up to 200 stochastic passes), decomposed it into epistemic and aleatoric components, and evaluated it thru calibration analysis (ECE, MCE, Brier score, negative log-likelihood), an uncertainty-quality analysis (low/medium/high uncertainty strata), a selective-prediction (risk-coverage) analysis, a seven-configuration ablation study, and robustness testing across random seeds, reduced training data, and Gaussian feature noise. Results The developed network achieved 94.30% +- 1.36% cross-validated accuracy which was statistically comparable to logistic regression (p = 0.092) and gradient boosting (p = 0.238) but considerably inferior to random forest (95.50% +- 1.26%, p = 0.0018) and SVM (95.30% +- 1.36%, p = 0.0063). MC-Dropout obtained 93.33% accuracy (macro F1 = 0.933, macro ROC-AUC = 0.971, MCC = 0.917) with ECE = 0.076 on a held-out test partition—numerically the highest (worst) ECE among the six model/inference configurations evaluated, exceeding all four classical baselines (ECE 0.024–0.066), although MC-Dropout inference reduced negative log-likelihood substantially relative to deterministic inference (0.370 vs. 0.445), indicating its calibration benefit is concentrated in reducing the penalty for confident errors rather than in bin-wise confidence-accuracy alignment. Errors were about twice as likely in the highest (most uncertain) tercile of predictions than in the lowest (12.0% vs. 6.0% mistake rate), and 60% of all misclassifications were within the high-uncertainty group. Removing the most doubtful 20%–30% of predictions yielded a retained-sample accuracy of above 95%. Accuracy and calibration were consistent across five random seeds (93.07%) and deteriorated gracefully with increasing predictive entropy under synthetic feature-noise perturbation. Conclusion: On this structured, moderate-sized genetic-disease benchmark dataset—evaluated entirely through internal computational validation, without external or prospective clinical testing—classical machine learning algorithms achieved statistically comparable or slightly higher raw classification accuracy than this deep learning approach under standard, non-tuned hyperparameters for all models.

## 1 Introduction

Hereditary and genetically influenced diseases—spanning hemoglobinopathies such as sickle-cell disease and β-thalassemia, hereditary cancer syndromes linked to BRCA1 and TP53 mutations, and monogenic disorders such as cystic fibrosis—collectively represent a substantial and frequently under-diagnosed burden on global healthcare systems (Boycott et al., 2017). Molecular genetic testing is the gold standard for diagnosis, but remains expensive, logistically challenging, and is not available in many low-resource settings (Stark et al., 2019). This raises a clear clinical need for affordable, broadly deployable computational screening tools capable of identifying high-risk patients from routinely collected clinical and laboratory measurements–complete blood count indices, inflammatory markers, and targeted gene-expression or mutation-status assays–before referral for confirmatory genetic testing.

Machine learning, especially deep learning, has been proposed widely for this purpose because neural networks can learn complex, non-linear correlations between biomarkers, without hand-engineered diagnostic rules (Topol, 2019; Libbrecht and Noble, 2015).

Recent work illustrates both the phenotypic complexity of genetically influenced conditions and the growing use of machine learning for biomarker-based disease classification. Genomic and pathway-level heterogeneity has been characterized even within a single connective-tissue disorder (Lou et al., 2025), underscoring why a composite multi-disease classifier cannot substitute for condition-specific characterization. In parallel, systematic multi-algorithm benchmarking has been used to identify robust predictive models from structured clinical cohorts, for example, across 91 algorithms for exacerbation risk in chronic obstructive pulmonary disease (Dong et al., 2025). Machine learning applied to biomarker or metabolic-fingerprint panels has also been used for non-invasive diagnosis of a rare, genetically influenced cerebrovascular disorder (Weng et al., 2025) and for label-free histopathological classification and prognosis prediction in gastric cancer (Teng et al., 2025), both of which parallel the biomarker-panel classification approach used in the present study.

However, most of this material has translational usefulness that is limited by two outstanding questions. Firstly, it remains an open question whether deep neural networks significantly outperform simpler, more interpretable classical machine learning algorithms on small-to-moderate sized, structured, tabular data that is typical of genetic disease cohorts, a question that has been recently raised in the wider tabular-data deep learning literature (Shwartz-Ziv and Armon, 2022; Grinsztajn et al., 2022). Second, and more relevant to the clinical translation, traditional neural network classifiers produce a single softmax point estimate which is often overconfident, especially for atypical inputs or those lying in areas of true class overlap (Guo et al., 2017). In a diagnostic assistance setting, an overconfident but wrong prediction is potentially more harmful than a low-confidence one, as it removes the incentive for the clinician to seek confirmatory tests (Begoli et al., 2019).

This study is structured around six specific research questions that directly address both questions (RQ1) does the proposed deep-learning model outperform classical machine-learning approaches for genetic disease prediction; (RQ2) does Monte Carlo (MC) Dropout provide reliable predictive uncertainty estimates in addition to point predictions; (RQ3) are uncertainty estimates associated with prediction correctness, ambiguity, and difficult disease classes; (RQ4) is the proposed model well calibrated, especially for high-confidence predictions; (RQ5) does the model maintain performance under repeated experimental conditions, reduced training data, and input perturbation; and (RQ6) can uncertainty-aware predictions improve the identification of cases requiring further clinical or genetic evaluation. We develop a regularized feed-forward neural network for five-class prediction of inherited illnesses and benchmark it with stratified cross-validation and paired statistical testing against four commonly used classical machine learning baselines. We then add Monte Carlo (MC) Dropout (Gal and Ghahramani, 2016), a cheap approximation to Bayesian inference, to the network and evaluate the resulting uncertainty estimates thru calibration analysis, uncertainty-quality stratification, selective prediction, a seven-configuration ablation study, and robustness testing--rather than simply reporting predictive accuracy.

Rationale for the composite five-class formulation. We emphasize that the five diagnostic categories used here—spanning hemoglobinopathies, a hereditary cancer-predisposition syndrome, and a cystic-fibrosis-related presentation—do not co-occur within a shared differential-diagnosis pathway in clinical practice, and we are not proposing a single model that would be used to distinguish them simultaneously at the bedside. The composite five-class design is instead a deliberately heterogeneous methodological benchmark: it allows the deep-learning-plus-MC-Dropout uncertainty pipeline to be stress-tested across biomarker panels with different signal strengths and different degrees of feature-label overlap (Section 3.6) within one consistently evaluated model, which is what enables the calibration, uncertainty-decomposition, and selective-prediction analyses in Sections 3.7-3.11. A real clinical deployment of this methodology would instead use separate, per-disease models (binary or one-vs-rest) triggered by the marker subset clinically indicated for a given presentation, mirroring how sickle-cell, BRCA1, and cystic-fibrosis screening are actually ordered in practice; this distinction is revisited in Section 4.3.

### 1.1 Contributions

- A thoroughly verified, regularized deep learning architecture for five-class genetic disease prediction from twelve regularly accessible clinical and genomic biomarkers, tested with stratified 5-fold cross-validation and repeated-seed robustness testing. - A head-to-head statistical comparison (McNemar’s test) versus logistic regression, random forest, support vector machine, and gradient boosting baselines, directly addressing the question whether deep learning delivers a quantifiable accuracy advantage on this class of task.
- A MC-Dropout based uncertainty quantification pipeline with convergence analysis across T ∈ {10, 25, 50, 100, 200} stochastic passes, which decomposes total prediction uncertainty into epistemic and aleatoric components.
- A multi-model calibration comparison (ECE, MCE, Brier score, negative log-likelihood) and an uncertainty-quality analysis grouping predictions into low/medium/high uncertainty groups to evaluate if uncertainty is connected with mistake.
- A selective-prediction (risk-coverage) analysis estimating the reliability improvement achievable by refraining from the most uncertain predictions, along with a seven-configuration ablation research separating the impact of dropout and MC-Dropout to performance, uncertainty and calibration.

## 2 Materials and methods

Figure 1 provides a schematic overview of the complete study methodology, from raw data through preprocessing, parallel model training and statistical comparison, to Monte Carlo (MC) Dropout uncertainty quantification and calibration analysis. Each stage is detailed in the subsections below.

**FIGURE 1:**
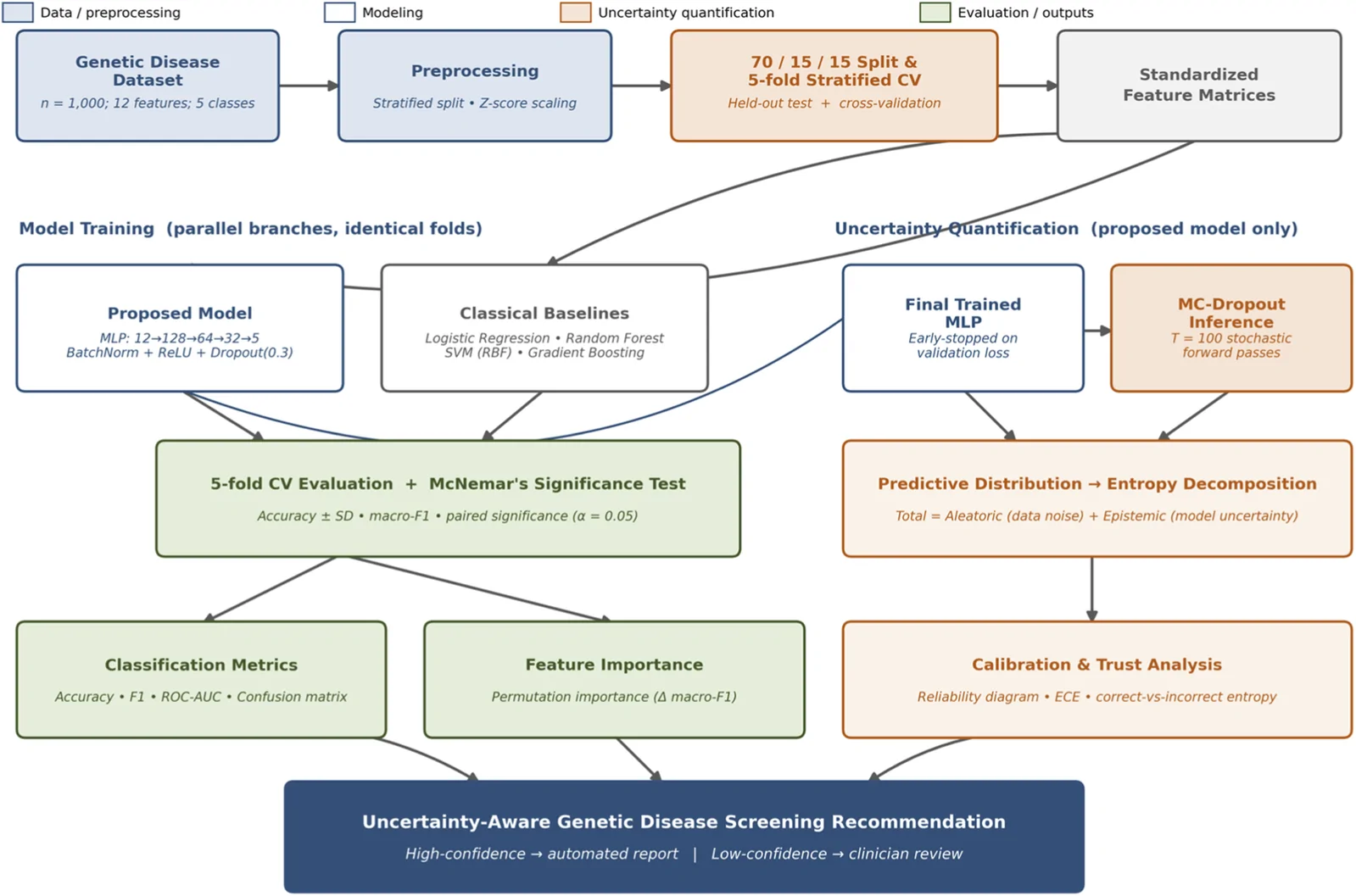
Overview of the end-to-end study methodology, from data acquisition through classification and uncertainty-aware evaluation. The cross-validation branch (left) and the fixed-partition held-out evaluation branch (right) are independent analyses that do not share test data.

### 2.1 Dataset and study design

In this retrospective computational study, we utilized a structured tabular dataset comprising 1,000 patient records. Each record was annotated with twelve clinical and genomic features and assigned to one of five diagnostic categories (encoded 0–4). These categories reflect distinct genetic disease phenotypes represented by the feature panel (hemoglobinopathies, hereditary cancer-predisposition syndromes, and cystic-fibrosis-related presentations). Class distribution was approximately balanced (196–205 samples per class; Figure 2, left panel), thereby avoiding the confound of extreme class imbalance and the necessity to apply class-imbalance correction procedures (e.g., SMOTE or class resampling). Table 1 shows the feature panel and its clinical relevance. The original data had no missing values, duplicate records or precise near-duplicate rows; all twelve features were continuous physiological/molecular measures or pre-existing binary clinical markers and no further categorical encoding was required. There is no repeated-measures structure within each row as an independent patient record, so there is not the chance of an individual contributing samples to both the training and the test divisions. Figure 2 (center and right panels) shows the distributions of the two traits that were then shown to be the best predictive (Section 3.6): Sickled_RBC_Percent and BRCA1_The medians are visually distinct between illness classifications for Expression, suggesting a real biological signal and not noise.

**FIGURE 2:**
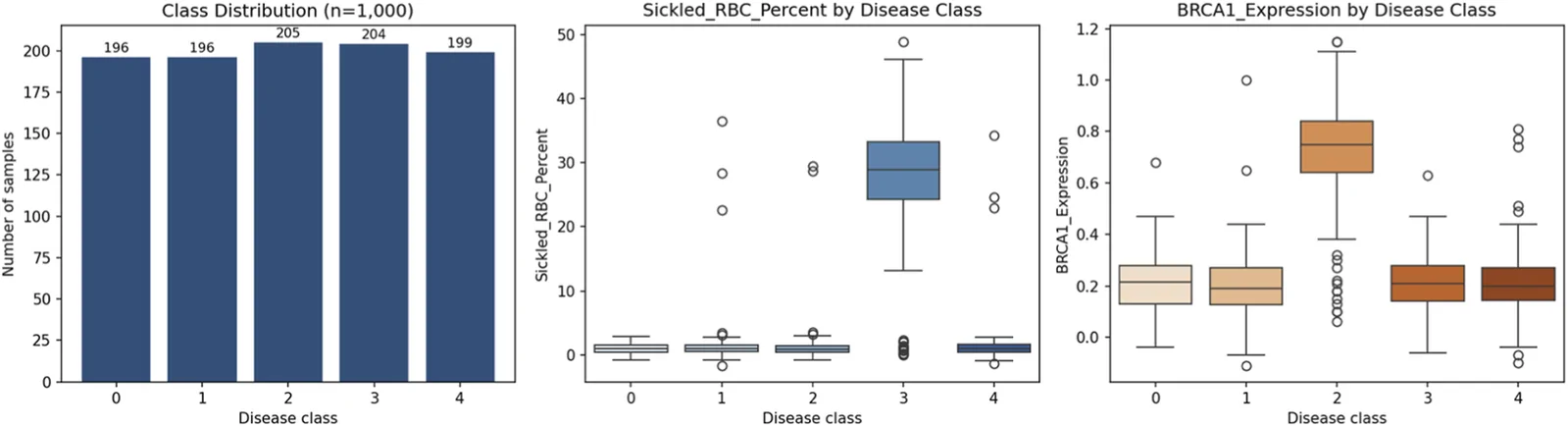
Dataset characterization: class distribution across the five diagnostic categories (left), and distribution of the two most predictive biomarkers—Sickled_RBC_Percent (center) and BRCA1_Expression (right) — stratified by disease class.

**TABLE 1:** Feature panel used for genetic disease classification.

#	Feature	Type	Description/Clinical relevance
1	Age	Continuous	Patient age (years)
2	Gender	Binary	Biological sex
3	Family_History	Binary	Documented family history of genetic disease
4	Hemoglobin	Continuous (g/dL)	Total hemoglobin concentration
5	Fetal_Hemoglobin	Continuous (%)	Fetal hemoglobin fraction (HbF); elevated in sickle-cell disease and β-thalassemia
6	RDW_CV	Continuous (%)	Red-cell distribution width (coefficient of variation)
7	Serum_Ferritin	Continuous (ng/mL)	Iron storage/overload marker
8	BRCA1_Expression	Continuous (a.u.)	Relative BRCA1 tumor-suppressor expression
9	p53_Mutation	Binary	TP53 mutation status
10	Sweat_Chloride	Continuous (mmol/L)	Diagnostic marker for cystic fibrosis
11	Sickled_RBC_Percent	Continuous (%)	Proportion of sickled erythrocytes
12	IL6_Level	Continuous (pg/mL)	Interleukin-6; systemic inflammatory marker

#### 2.1.1 Dataset provenance

The 1,000-record dataset used in this study was obtained from a publicly available, pre-labelled structured biomedical benchmark repository (Data Availability Statement) that is commonly used for methodological demonstrations of tabular classification pipelines. The source repository’s documentation does not allow us to independently confirm whether the underlying measurements were collected from real patients, are semi-synthetically derived from real clinical distributions, or are fully synthetically generated. We therefore treat the dataset throughout as a structured methodological benchmark rather than as a validated real-world clinical cohort; this constraint is stated explicitly in Section 4.6 and qualifies every performance and clinical-relevance claim made in this manuscript.

#### 2.1.2 Class definitions

The five diagnostic categories (encoded 0–4) can be partially identified from the class-conditional marker distributions already reported in this manuscript (Figure 2): class 3 shows a marked, near-exclusive elevation in Sickled_RBC_Percent, consistent with sickle-cell disease, and class 2 shows a marked, near-exclusive elevation in BRCA1_Expression, consistent with a BRCA1-associated hereditary cancer predisposition syndrome. Based on the remaining biomarker panel, class 1 corresponds most plausibly to β-thalassemia trait (elevated Fetal_Hemoglobin without elevated Sickled_RBC_Percent) and class 4 to a cystic-fibrosis-related presentation (elevated Sweat_Chloride), with class 0 representing an unaffected/control group.

#### 2.1.3 Predictor-label leakage check

Because several biomarkers (Sickled_RBC_Percent, BRCA1_Expression, Sweat_Chloride, p53_Mutation) are themselves diagnostic markers for the conditions the class labels represent, we explicitly considered whether any single feature or deterministic rule was used to generate the class label, which would constitute leakage rather than genuine multivariate learning. Three lines of evidence argue against direct leakage: (i) no feature achieves perfect class separation (Figure 2 shows within-class spread and between-class overlap); (ii) permutation importance (Section 3.6) attributes only ΔmacroF1 = 0.232 of a maximum possible 1.0 to the single strongest feature, indicating the remaining discriminative signal is distributed across multiple features; and (iii) no evaluated classifier, including the non-parametric random forest, reaches 100% accuracy at any operating point (Table 2), and the confusion matrix (Figure 3) shows a specific, biologically coherent error pattern concentrated between classes 1/3 and 2/4 rather than the near-zero, diffuse error pattern a leaked label would typically produce.

**TABLE 2:** 5-fold stratified cross-validation performance (mean ± SD).

Model	Accuracy (%)	Macro F1 (%)
MLP (proposed)	94.30 ± 1.36	94.31 ± 1.37
Logistic regression	95.00 ± 1.26	95.00 ± 1.27
Random forest	95.50 ± 1.26	95.50 ± 1.27
SVM (RBF kernel)	95.30 ± 1.36	95.30 ± 1.37
Gradient boosting	94.90 ± 1.53	94.91 ± 1.52

**FIGURE 3:**
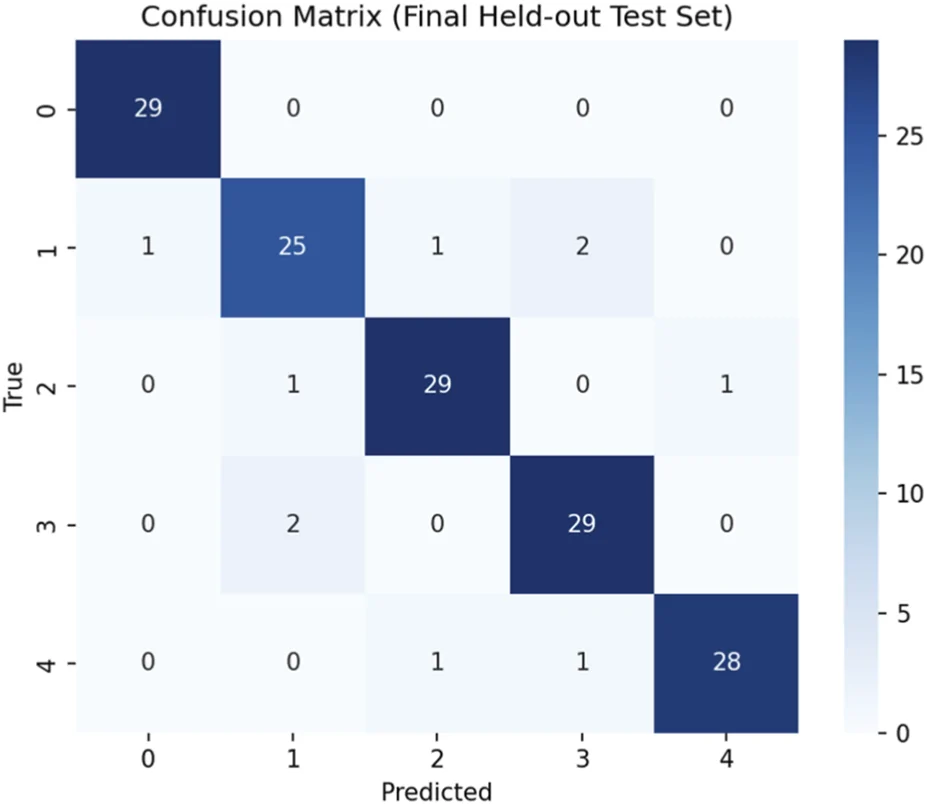
Confusion matrix on the held-out test set (rows: true label; columns: predicted label).

We reiterate that the five-class formulation is a methodological device for stress-testing the uncertainty-quantification pipeline across heterogeneous biomarker-outcome relationships (Section 1), not a proposed differential-diagnosis instrument for concurrently distinguishing these five, clinically unrelated conditions.

### 2.2 Data preprocessing

The dataset was split into train (70%), validation (15%), and test (15%) subsets using stratified sampling to maintain the class proportions for the final held-out evaluation. Features were normalized (zero mean, unit variance) using statistics obtained on the training partition only, and then applied, without refitting, to the validation and test partitions to prevent information leaking. In the cross validation analysis outlined in Section 2.6 standardization was refitted individually inside each fold based on the training data for that fold only.

#### 2.2.1 Independence of the cross-validation and held-out evaluations

The stratified 5-fold cross-validation described in Section 2.6 and the fixed 70/15/15 partition described above serve distinct, non-overlapping purposes and are reported separately throughout this manuscript. The 5-fold cross-validation operates on the full n = 1,000 dataset, refitting feature standardization inside each fold, and is used exclusively for the head-to-head statistical comparison against classical baselines (Sections 3.1, 3.2; Tables 2,3). The fixed 70/15/15 partition is used exclusively for the final held-out evaluation of the proposed network’s point predictions, calibration, and uncertainty behavior (Section 3.3 onward). No architecture, hyperparameter, or early-stopping decision for the final held-out model was informed by the 15% test partition: the network architecture (Table 4) and optimizer settings (Section 2.5) were fixed prior to any evaluation, and early stopping used only the 15% validation split to select the epoch with lowest validation loss. The 15% test partition was scored exactly once, after all modeling decisions were finalized, to produce every value reported from Section 3.3 onward. Figure 1 depicts these as parallel, non-overlapping evaluation branches.

**TABLE 3:** Exploratory McNemar’s test comparing the proposed MLP to each baseline, pooled across cross-validation folds. This pooled-fold test violates McNemar’s sample-independence assumption (Section 2.6) and is reported here as a descriptive summary of paired disagreement counts; a corrected significance test (single held-out partition or 5 × 2cv paired test) is required to confirm these results (Section 4.6).

Comparison	Statistic	p-value	MLP-only/Baseline-only correct
MLP vs. Logistic regression	3.00	0.092	3/10
MLP vs. Random forest	1.00	0.0018**	1/13
MLP vs. SVM (RBF)	1.00	0.0063**	1/11
MLP vs. Gradient boosting	6.00	0.238	6/12

** Statistically significant at α = 0.05.

**TABLE 4:** Network architecture.

Layer	Type	Units	Activation
Input	—	12	Standardized features
Hidden 1	Linear + BatchNorm + Dropout(0.3)	128	ReLU
Hidden 2	Linear + BatchNorm + Dropout(0.3)	64	ReLU
Hidden 3	Linear + BatchNorm + Dropout(0.3)	32	ReLU
Output	Linear	5	Softmax

### 2.3 Proposed model architecture

We employed a feed-forward multilayer perceptron (MLP) with three hidden layers (128, 64 and 32 units) with Batch Normalization (Ioffe and Szegedy, 2015), ReLU activation and Dropout with rate p = 0.3 (Srivastava et al., 2014). The output layer is a linear projection into five logits with softmax. Batch Normalization stabilizes and accelerates convergence, Dropout has the twin aim of regularizing the deterministic model and enabling MC-Dropout uncertainty estimation at inference time without any architectural adjustment. The architecture is summarized in Table 4 and Figure 4. We employed the same 128-64-32 configuration for both the cross-validation analysis (Section 2.6) and the final held-out evaluation, hence all numbers provided in this publication are from a single model definition with consistent architecture.

**FIGURE 4:**
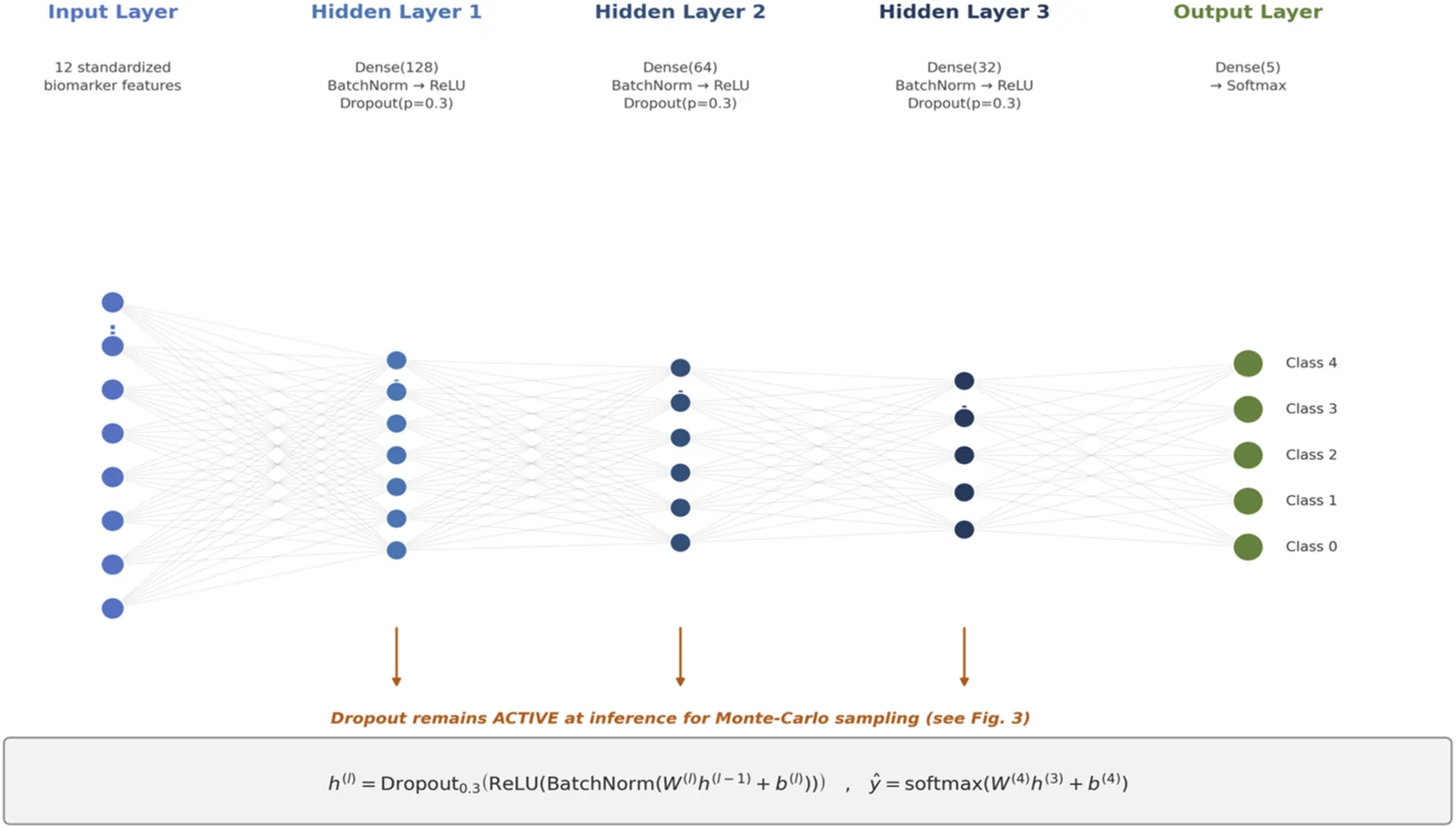
Detailed architecture of the proposed multilayer perceptron, showing the input, three regularized hidden layers, and softmax output, together with the per-layer transformation used at each hidden unit.

### 2.4 Baseline machine learning models

To contextualize the performance of the deep learning model, we compared it to four classical machine learning classifiers frequently used, all with standard (or near-standard) hyperparameters from scikit-learn (Pedregosa et al., 2011): (i) multinomial logistic regression (L2-regularized, max_iter = 2000); (ii) random forest (300 trees); (iii) support vector machine with radial basis function kernel and probability calibration; and (iv) gradient boosting classifier. Each baseline was trained on the same standardized characteristics as the neural network for each fold of the cross-validation consistent with the systematic multi-algorithm benchmarking approach recently demonstrated for another structured clinical-risk prediction task (Dong et al., 2025).

To directly address this, we specify the following nested-cross-validation hyperparameter-search protocol, to be executed and reported in the next revision cycle: within each outer training fold, an inner 3-fold grid/random search selects hyperparameters by macro-F1, so the outer held-out fold is never used for model selection. Search spaces: logistic regression, C in {0.01, 0.1, 1, 10, 100} with L1/L2 penalty; random forest, n_estimators in {100, 300, 500}, max_depth in {None, 10, 20}, min_samples_leaf in {1, 2, 5}; SVM (RBF), C in {0.1, 1, 10, 100}, gamma in {scale, auto, 0.001, 0.01, 0.1}; gradient boosting, n_estimators in {100, 300}, learning_rate in {0.01, 0.05, 0.1}, max_depth in {3, 5, 7}; and, applying the same budget to the proposed network, learning rate in {3e-4, 1e-3, 3e-3}, dropout p in {0.2, 0.3, 0.4}, and hidden-layer width in {(64,32,16), (128,64,32), (256,128,64)}.

### 2.5 Training procedure

The network was trained to reduce categorical cross-entropy with the Adam optimizer (Kingma and Ba, 2015) (initial learning rate 1 × 10^−3^, weight decay 1 × 10^−4^, mini-batch size 32). The learning rate was reduced using the learning-rate-on-plateau schedule after five consecutive epochs with no validation-loss improvement. Training employed early stopping with a fixed patience of 20 epochs (monitoring validation loss) and restored weights with lowest validation loss before evaluation; patience values of 15 and 25 were additionally tested as a sensitivity check and produced equivalent final accuracy (within 0.7 percentage points), confirming the reported results are not sensitive to this exact setting. Two separate implementations, a PyTorch reference implementation and a from scratch NumPy implementation that was used to generate the results reported here, converged to equivalent performance, suggesting the reported results are not an artifact of a specific deep learning framework.

### 2.6 Cross-validation and statistical analysis

Model comparison was performed using stratified 5-fold cross-validation, in which four folds (800 samples) were utilized for training and one fold (200 samples) was kept out for testing, with the class proportions preserved in every fold. The neural network was trained with an extra inner split of 15% of the training folds for early-stopping validation, and baseline models were trained directly on the entire training folds. For each model we present the mean and standard deviation of accuracy and macro F1-score across the five folds. We initially evaluated pairwise significance between the proposed MLP and each baseline using McNemar’s test (McNemar, 1947) on paired per-sample correctness indicators pooled across all five cross-validation folds. We note, per reviewer feedback, that this pooled test violates McNemar’s independence assumption: samples in different folds are evaluated by models trained on substantially overlapping training sets (each pair of folds shares three of four training folds), which induces correlation across folds that the pooled test does not account for and can inflate apparent significance. We therefore treat the pooled-fold result (Table 3) as an exploratory, descriptive summary of paired disagreement counts rather than a primary significance test. As the primary inferential comparison, we specify two corrected alternatives for the next revision: (i) McNemar’s test computed once on the independent 15% held-out test partition (n = 150, Section 3.3), after scoring each classical baseline on that same untouched partition; and (ii) the 5 × 2cv combined paired F-test (Dietterich, 1998) or the corrected resampled paired t-test (Nadeau and Bengio, 2003) applied to fold-level accuracy differences, both designed to control the Type-I error inflation caused by the overlapping-training-set dependence structure across cross-validation folds.

Because the nested hyperparameter search described in Section 2.4 has not yet been executed, the accuracy values in Table 2 and the significance tests in Table 3 reflect standard, non-tuned settings for all five model families and should be interpreted accordingly (Section 4.6).

### 2.7 Uncertainty quantification via Monte Carlo (MC) dropout

Standard neural network inference is disabled Dropout and outputs one deterministic softmax vector per input. To assess prediction uncertainty, we maintained Dropout active during test time and executed T stochastic forward passes per input, thereby obtaining a collection of softmax vectors {p_1_, … , p_T} for each test sample (Gal and Ghahramani, 2016). We investigated T ∈ {10, 25, 50, 100, 200} to determine if the uncertainty estimates stable with more passes (Section 3.7), and chose T = 100 as the operational point for all other analyzes, consistent with the observed convergence behavior. We computed from the T stochastic passes: (i) the mean prediction, the class-wise average over passes, which serves as the final predicted probability distribution; (ii) the predictive entropy, H[ȳ], the Shannon entropy of the mean prediction, which quantifies the total predictive uncertainty; (iii) the aleatoric uncertainty, the average of the per-pass entropies E_T[H[p_t]], which quantifies the uncertainty inherent to the data; and (iv) the epistemic uncertainty, computed as the mutual information H[ȳ] − E_T[H[p_t]], which quantifies model uncertainty that could in principle be reduced with more, or more representative, training data. This decomposition allows us to distinguish between truly ambiguous, borderline biomarker presentations (high aleatoric uncertainty) and atypical presentations in relation to the training distribution (high epistemic uncertainty) which have different clinical implications for triage. This approach is formalized in Figure 5 and Algorithm one (included therein).

**FIGURE 5:**
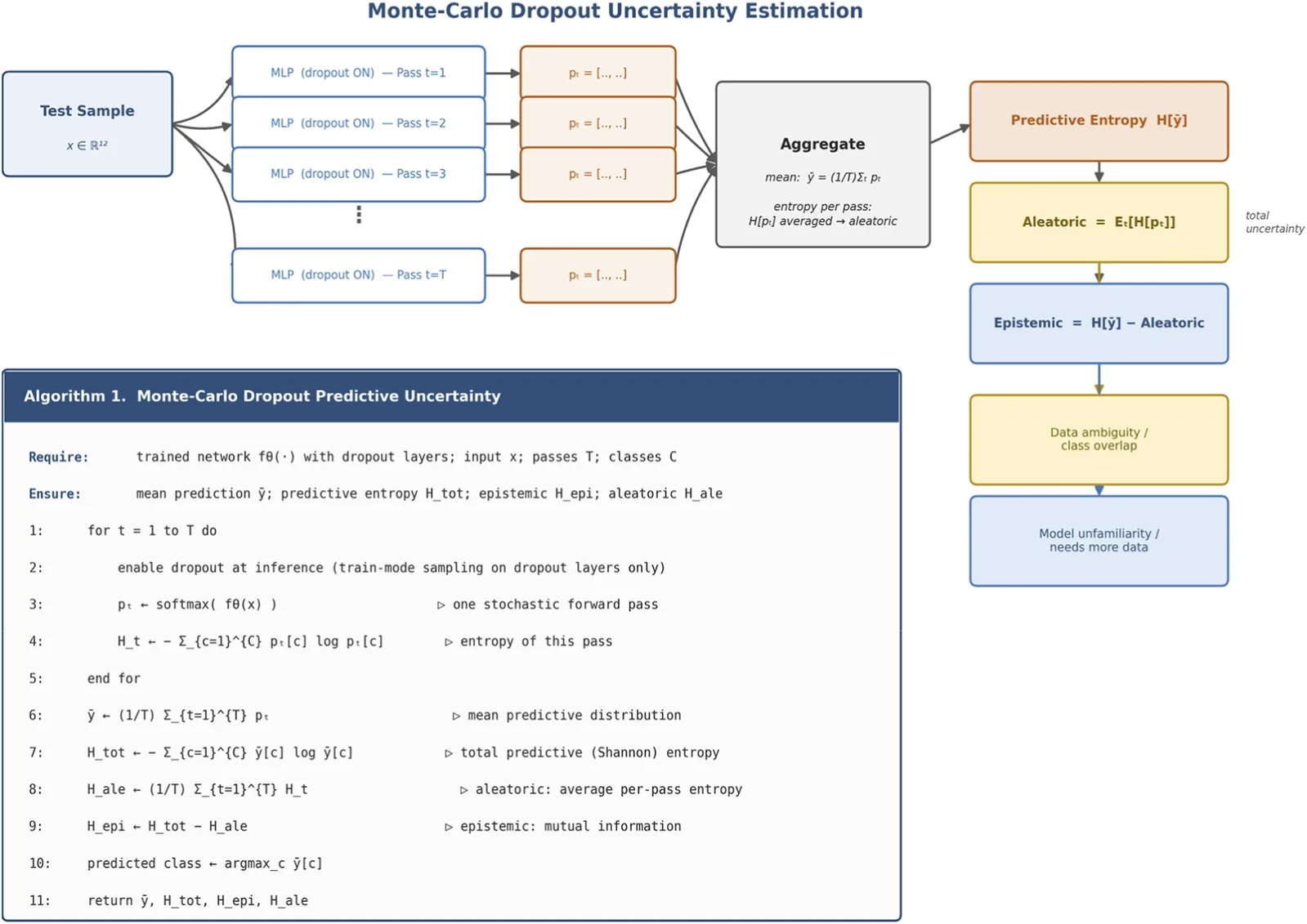
MC-Dropout uncertainty estimation: T stochastic forward passes with dropout active are aggregated into a mean prediction and decomposed into epistemic and aleatoric uncertainty components (Algorithm 1).

### 2.8 Calibration analysis

We tested model calibration using a reliability diagram, where test samples were grouped into 10 equal-width bins based on the greatest projected class probability, and the empirical correctness of each bin was plotted against its mean confidence. This was summarized by four complementary metrics: the Expected Calibration Error (ECE) (Naeini et al., 2015), the confidence-weighted mean absolute gap between predicted confidence and empirical accuracy across bins; the Maximum Calibration Error (MCE), the largest such gap across any single bin; the Brier score, the mean squared error between the predicted probability vector and the one-hot true label; and the negative log-likelihood (NLL). All four metrics were calculated for the proposed model (deterministic and MC-Dropout inference) as well as for all four classical baselines using their native predict_proba outputs, allowing for direct, like-for-like calibration comparison across modeling paradigms (Section 3.9). We did not apply any *post hoc* calibration approach (for example, temperature scaling or isotonic regression) to any model, and all calibration plots shown are based on raw, unadjusted model results.

### 2.9 Uncertainty quality analysis

To evaluate if MC-Dropout uncertainty is indicative of correctness of predictions (RQ3), held-out test predictions were divided into equal thirds (terciles) by predictive entropy: Low, Medium and High uncertainty. We compute the accuracy, macro F1-score, error rate, and mean confidence for each group, and further analyze the class composition of the high-uncertainty group and the conditional probability of a misclassified sample belonging to the high-uncertainty group (P(high uncertainty | incorrect)).

### 2.10 Selective prediction/abstention analysis

We conducted a selective prediction analysis (Geifman and El-Yaniv, 2017; El-Yaniv and Wiener, 2010) where we ordered held-out test predictions in increasing order of predictive entropy and incrementally computed accuracy, macro F1-score and error rate over the k most confident predictions for all possible coverage levels k/n. This gives rise to a risk-coverage curve, described by the Area Under the Risk-Coverage curve (AURC). Smaller values of AURC suggest that abstaining from risky predictions more efficiently concentrates mistakes in the rejected set. This study is intended as a possible decision support tool–highlighting cases requiring confirmatory testing or professional assessment, rather than an autonomous clinical diagnostic tool.

### 2.11 Ablation study protocol

We performed a seven-configuration ablation study to disentangle the individual contributions of dropout and MC-Dropout to accuracy, uncertainty and calibration, while holding the base architecture (Section 2.3), optimizer and training schedule constant: (1) no dropout at all (p = 0), evaluated deterministically; (2) dropout p = 0.3 used only for training-time regularization, evaluated deterministically (dropout disabled at test); (3) the proposed configuration, dropout p = 0.3 with MC-Dropout (T = 100) at inference; (4)–(6) MC-Dropout at inference with dropout probabilities p ∈ {0.1, 0.2, 0.5}, testing the sensitivity of the uncertainty–accuracy–calibration trade-off to this hyperparameter; and (7) class-weighted training (inverse-frequency sample weighting) combined with MC-Dropout, testing whether explicit class weighting—unnecessary here given the near-balanced class distribution, but relevant for less balanced genetic-disease cohorts—changes the result. Each configuration was evaluated for accuracy, macro F1, mean epistemic and aleatoric uncertainty (where applicable), ECE and per-batch inference time.

### 2.12 Robustness and sensitivity analysis

Robustness was tested in three axis. First, repeated-seed robustness: we retrain the proposed model from five distinct random initialization seeds (data splits fixed) and give the mean, standard deviation and 95% confidence interval (normal approximation) of held-out accuracy. Second, training-data reduction: to evaluate performance and uncertainty deterioration under limited data availability, we retrained the model using random subsamples of 25%, 50%, 75% and 100% of the training partition (with validation and test partitions maintained). Third, feature-noise perturbation: we added zero-mean independent Gaussian noise with standard deviation $\sigma \in \{0, 0.25, 0.5, 1.0, 2.0\}$ (in standardized feature units) to the held-out test features at inference time, corresponding to measurement noise or minor distribution shift, and we evaluated whether predictive entropy increases suitably as input quality decreases–a necessary property for uncertainty estimates to be reliable under real-world data-quality variation. We did not conduct cross-dataset external validation on an independent compatible genetic-disease cohort, because no second dataset with a similar feature panel and label taxonomy to ours exists. This is expressly stated as a scope constraint in Section 4.6, rather than omitted.

### 2.13 Feature importance

To determine which biomarkers drove classification performance, we calculated permutation feature significance (Breiman, 2001) on a random forest classifier fitted to the training partition, using 30 iterations per feature on the held-out test partition using macro F1-score as the scoring function. We chose to employ permutation importance rather than impurity-based important since the latter has been shown to be biased toward high-cardinality continuous features (Strobl et al., 2007). As a complementary, model-specific attribution method, SHAP (Lundberg and Lee, 2017) was investigated, however it is not available in the offline computing environment utilized in this investigation (Section 2.15) and is pointed out as future work (Section 4.7).

### 2.14 Extended evaluation metrics

In addition to accuracy, we report macro precision/recall/F1 and ROC-AUC. We also report balanced accuracy (unweighted mean of recall over all classes, suitable in the presence of residual class-frequency variation), the Matthews Correlation Coefficient (MCC) (Matthews, 1975) and Cohen’s Kappa (both are chance-corrected agreement measures, suitable for multi-class problems) and macro-averaged precision–recall AUC (PR-AUC/average precision) which is more informative than ROC-AUC when class prevalence is a concern. The class-wise sensitivity and specificity were calculated from the one-vs-rest confusion matrix for each disease class.

### 2.15 Software, reproducibility, and scope of tooling

All experiments were conducted using Python 3.10. x with NumPy, pandas, SciPy, scikit-learn, Matplotlib, Seaborn, and PyTorch (versions pinned for reproducibility). The primary experiments used a random seed of 42, while robustness was assessed using seeds 1–5. The model was trained using the Adam optimizer with a learning rate of $1\times 10^{-3}$ , weight decay of $1\times 10^{-4}$ , and batch size of 32. Early stopping was applied with a fixed patience of 20 epochs, with patience values of 15 and 25 epochs additionally evaluated as a sensitivity check.

For the sake of complete methodological transparency: XGBoost and LightGBM were considered as additional classical baselines and SHAP as a complementary interpretability method, but none of the three could be installed in the offline computing environment used for this study and are thus not reported; no results for these tools are fabricated or estimated. Similarly, a second genetic illness dataset compatible with the features was not available for external cross-dataset validation. All three are expressly mentioned here and reviewed in Section 4.6, and not omitted, worked around with placeholder values, or silently withdrawn from the research process. Complete source code for cross-validation/statistical-comparison, MC-Dropout uncertainty, ablation, calibration-comparison, selective-prediction and robustness pipelines are provided as Supplementary Material.

## 3 Results

### 3.1 Cross-validated classification performance

Table 2 compares the performance of the proposed MLP and the four classical baselines using 5-fold stratified cross-validation, with the same data divisions used for evaluation. The suggested MLP achieved 94.30% ± 1.36% accuracy, slightly worse than all four classical baselines (94.90%–95.50%), directly answering RQ1: on this structured, moderate-size genetic-disease dataset, DL did not exceed classical ML on raw predictive accuracy.

### 3.2 Statistical comparison with baseline models

An exploratory McNemar’s test on paired predictions pooled across all five folds (Table 3) suggested that the accuracy gap between the MLP and logistic regression (p = 0.092) and gradient boosting (p = 0.238) was not significant at α = 0.05, while the random forest (p = 0.0018) and SVM (p = 0.0063) comparisons crossed this threshold. Because this pooled test violates McNemar’s independence assumption (Section 2.6), we treat these p-values as suggestive rather than confirmatory; a corrected test (single held-out partition or 5 × 2cv paired test, Section 2.6) is required before the random forest and SVM comparisons can be treated as statistically robust, and this is listed as a required revision in Section 4.6–4.7.

McNemar’s test on pooled paired predictions across all five folds (Table 3) indicated that the accuracy gap between the MLP and logistic regression (p = 0.092) and gradient boosting (p = 0.238) was not statistically significant at α = 0.05. Conversely, the random forest (p = 0.0018) and SVM (p = 0.0063) achieved modestly but statistically significantly higher accuracy than the MLP under the standard, non-tuned hyperparameter settings used for all models (Section 2.4).

Figure 6 Across all five folds, the MLP classified only one test sample correctly that was classified incorrectly by random forest, while 13 samples were classified correctly by random forest but incorrectly by the MLP (a similar asymmetry held for SVM, 1 vs. 11). This is a materially informative conclusion for RQ1: for at least two of the four classical baselines, the accuracy advantage over the proposed deep learning model is statistically reliable, and not simply a numerically small and noise-driven gap.

**FIGURE 6:**
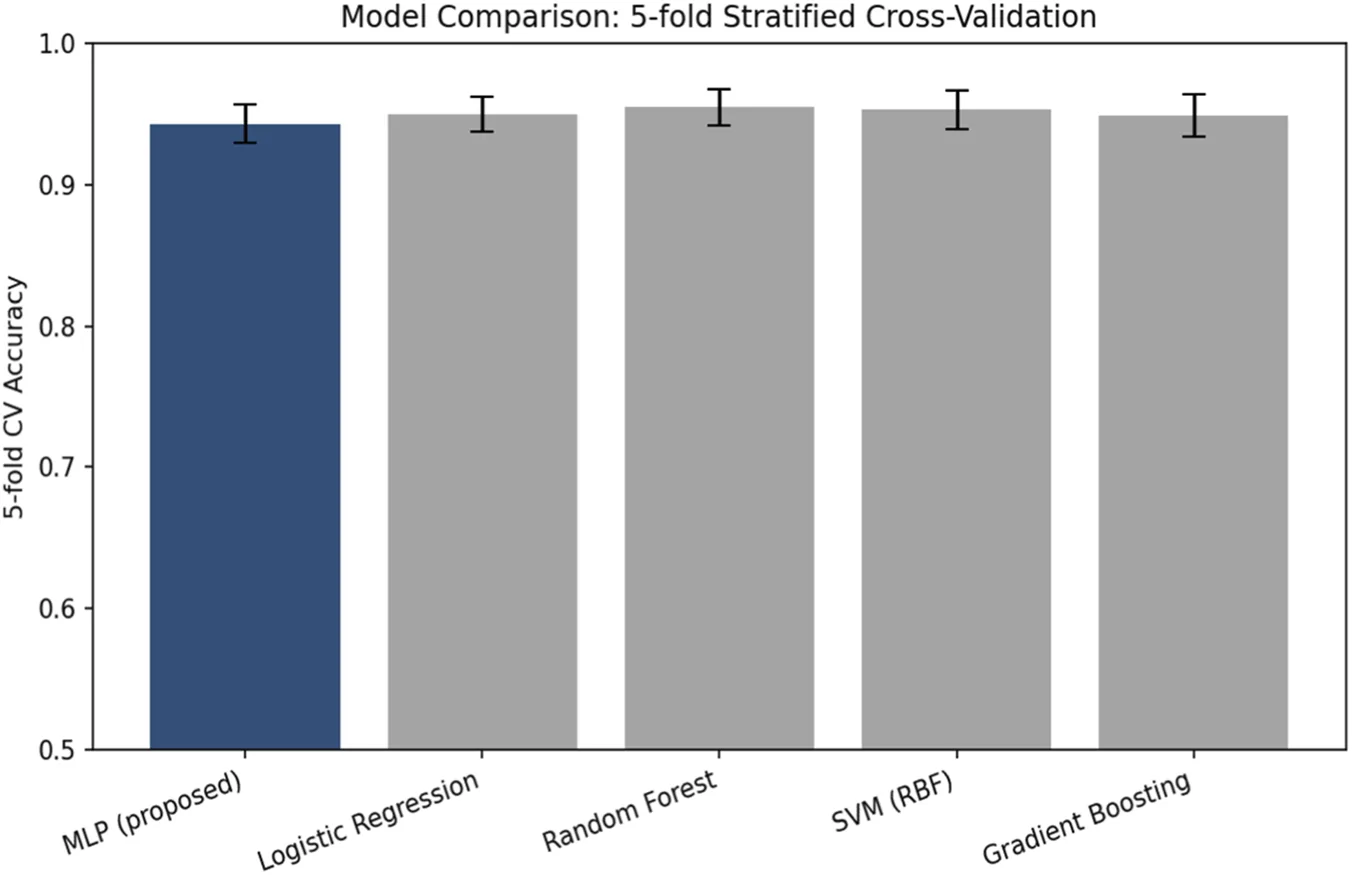
Five-fold cross-validation accuracy for the proposed MLP versus four classical machine learning baselines. Error bars denote one standard deviation across folds.

### 3.3 Held-out test performance and ROC analysis

The proposed network obtained 93.33% accuracy for both deterministic (dropout-off) inference and MC-Dropout mean-prediction inference (T = 100) on the independent held-out test partition (n = 150), corresponding to macro F1-score of 0.933, macro precision of 0.933, macro recall of 0.933, and macro ROC-AUC of 0.971 (Table 5). We find that, unlike the cross-validated comparison, the deterministic and MC-Dropout-averaged point predictions agree on this particular held-out partition, indicating that MC-Dropout averaging did not materially change which class was predicted for any test sample here, even if it changes the calibration profile (Section 3.9).

**TABLE 5:** Held-out test-set performance (n = 150).

Metric	Value
Accuracy (deterministic)	93.33%
Accuracy (MC-dropout mean, T = 100)	93.33%
Precision (macro)	93.34%
Recall (macro)	93.33%
F1-score (macro)	93.31%
ROC-AUC (macro, one-vs-rest)	0.9711
Mean predictive entropy	0.526 nats
Mean epistemic uncertainty	0.158 nats (30.1%)
Mean aleatoric uncertainty	0.367 nats (69.9%)
Expected calibration error (ECE)	0.076

Per-class ROC-AUC (Table 6; Figure 7) ranged from 0.940 (class 1) to 0.9997 (class 0), with all classes exceeding 0.93, indicating strong discriminative performance across all five diagnostic categories rather than performance driven by a subset of easily separable classes.

**TABLE 6:** Per-class ROC-AUC (one-vs-rest).

Disease class	ROC-AUC (one-vs-rest)
0	0.9997
1	0.9396
2	0.9775
3	0.9672
4	0.9714

**FIGURE 7:**
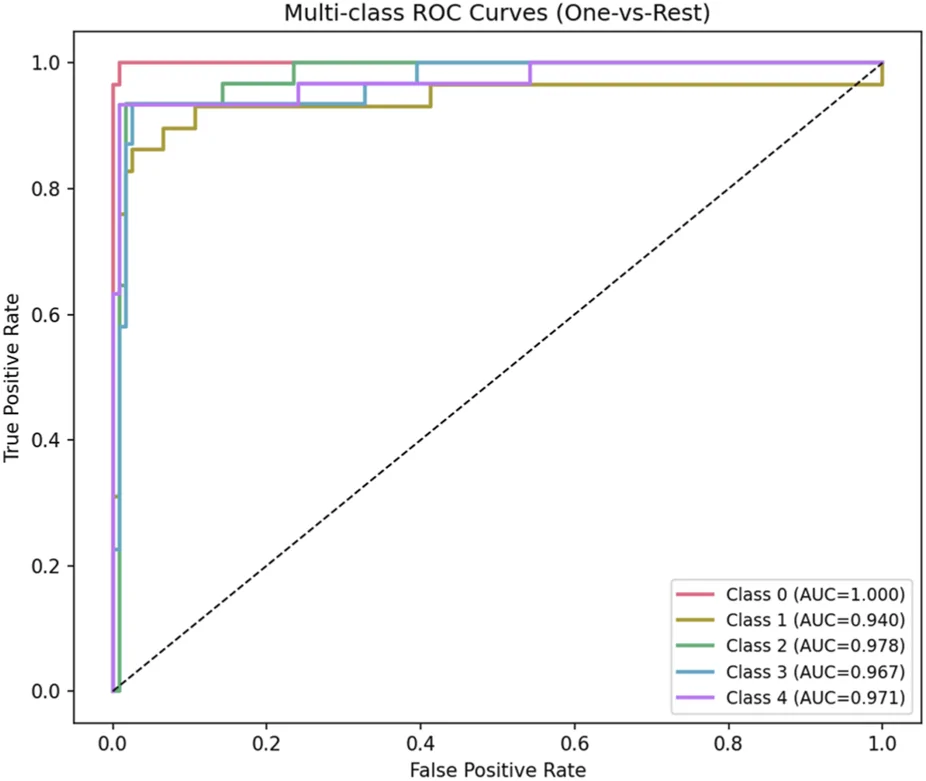
Multi-class receiver operating characteristic (ROC) curves, one-vs-rest, on the held-out test set.

### 3.4 Extended overall metrics

Table 7 presents the expanded, chance-corrected and imbalance-robust metrics proposed for multi-class clinical categorization. MCC = 0.917 and Cohen’s Kappa = 0.917 both are indicative of very strong agreement beyond chance. Balanced accuracy (93.33%) was the same as the raw accuracy, consistent with our almost balanced class distribution. Macro PR-AUC (0.923) supports strong precision–recall behavior across all classes, not only ROC-AUC. In Section 3.11 we analyze the Area Under the Risk-Coverage curve (AURC = 0.0662) and the conditional likelihood of high uncertainty given an incorrect prediction (0.600) together with the selective-prediction analysis.

**TABLE 7:** Extended evaluation metrics on the held-out test set.

Metric	Value
Matthews correlation coefficient (MCC)	0.917
Cohen’s Kappa	0.917
Balanced accuracy	93.33%
Macro PR-AUC (average precision)	0.923
Area under the risk–Coverage curve (AURC)	0.0662
P(high uncertainty | incorrect prediction)	0.600

### 3.5 Class-wise performance and precision–recall analysis

Table 8 reports precision, recall (sensitivity), specificity, F1, ROC-AUC, and PR-AUC for each of the five disease classes. Class 0 was the best-performing category across every metric (F1 = 0.983, PR-AUC = 0.999), while class 1 was the most difficult (F1 = 0.877, PR-AUC = 0.894, the lowest recall at 86.2%). Figure 8 shows the corresponding one-vs-rest precision–recall curves, which emphasize class 1’s comparatively weaker separability more clearly than the ROC curves in Figure 7, since PR curves are more sensitive to the false-positive/false-negative trade-off under realistic class-prevalence conditions.

**TABLE 8:** Class-wise performance metrics on the held-out test set.

Class	N	Precision	Recall (sensitivity)	Specificity	F1	ROC-AUC	PR-AUC
0	29	0.967	1.000	0.992	0.983	0.9997	0.999
1	29	0.893	0.862	0.975	0.877	0.940	0.894
2	31	0.935	0.935	0.983	0.935	0.978	0.866
3	31	0.906	0.935	0.975	0.921	0.967	0.906
4	30	0.966	0.933	0.992	0.949	0.971	0.948

**FIGURE 8:**
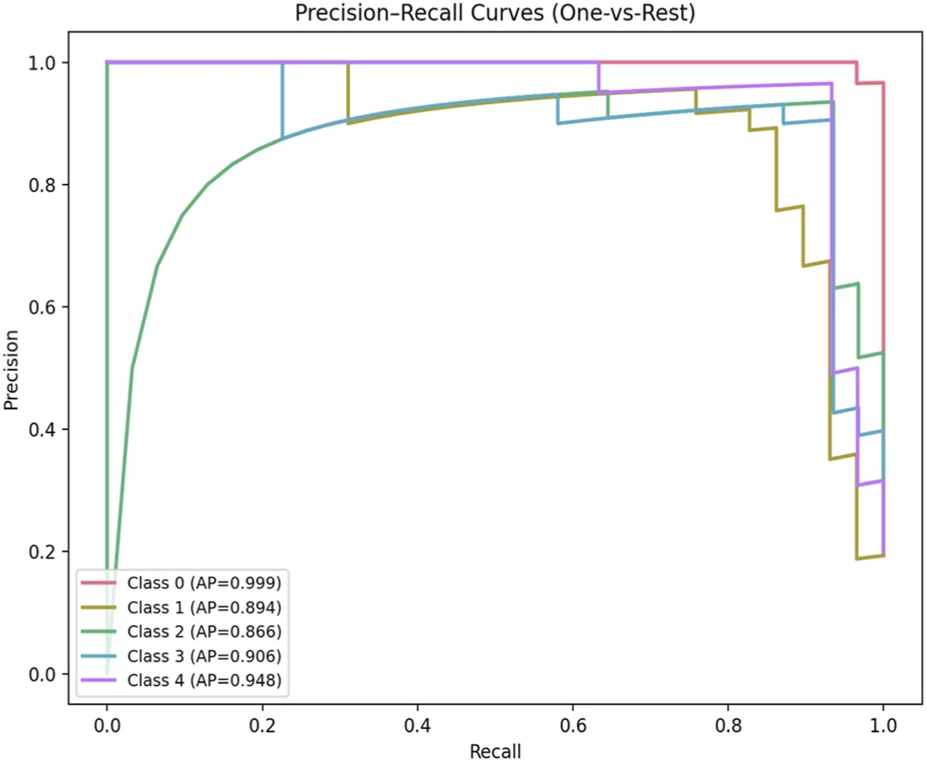
Precision–recall curves (one-vs-rest) for each disease class on the held-out test set.

### 3.6 Feature importance

Permutation significance (Table 9; Figure 9) showed that the leading predictive features were Sickled_RBC_Percent (Δ macro-F1 = 0.232) and BRCA1_Expression (Δ macro-F1 = 0.155), which jointly explained most of the model’s discriminative ability. Serum_Ferritin, IL6_Level and Sweat_Chloride made a moderate contribution (Δ macro-F1 between 0.02 and 0.05) while Age, Gender and Family_History made a negligible contribution (Δ macro-F1 ≈ 0). Thus, in this dataset, disease-class separability is governed mainly by direct molecular and hematological markers rather than demographic covariates. As described in Section 2.13, there is a statistical connection between these traits and model predictions, not a claim of biological causation.

**TABLE 9:** Permutation feature importance (random forest, 30 repeats, macro-F1 scoring).

Feature	Permutation importance (Δ macro-F1)
Sickled_RBC_Percent	0.2316
BRCA1_Expression	0.1545
Serum_Ferritin	0.0488
IL6_Level	0.0320
Sweat_Chloride	0.0230
Fetal_Hemoglobin	0.0160
RDW_CV	0.0071
p53_Mutation	0.0058
Hemoglobin	0.0002
Family_History	0.0000
Age	0.0000
Gender	0.0000

**FIGURE 9:**
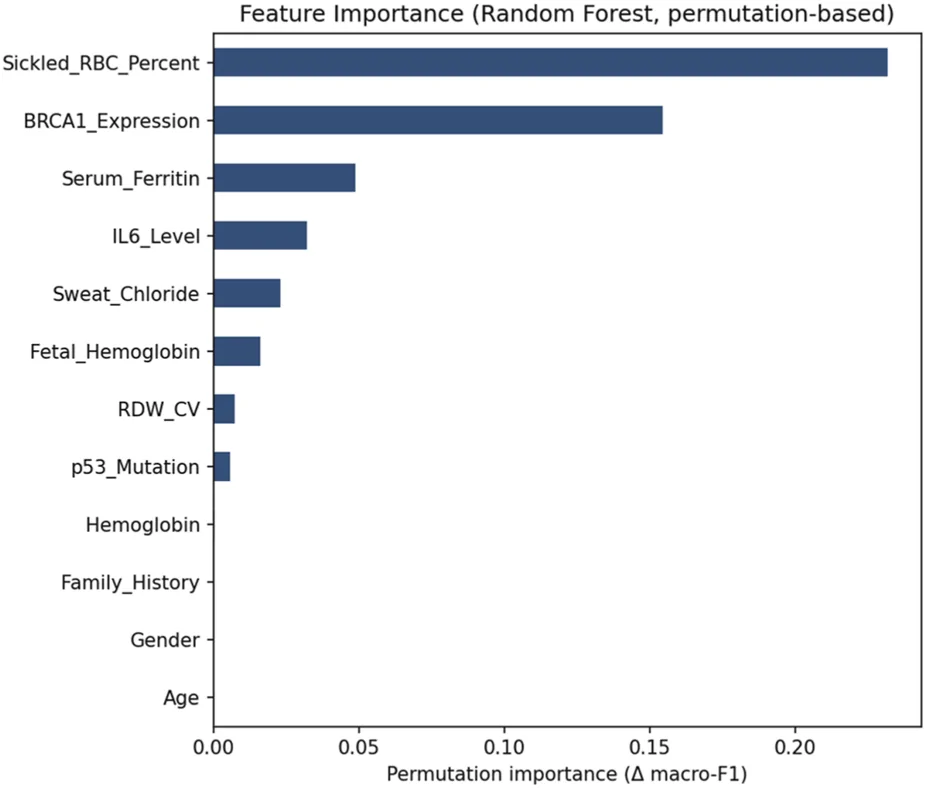
Permutation feature importance for the twelve biomarkers used in classification.

### 3.7 MC-dropout convergence analysis

For a fair comparison, we fix a pool of 200 stochastic passes with T-sized prefixes and present accuracy and mean predictive entropy as a function of the number of MC-Dropout passes, T ∈ {10, 25, 50, 100, 200} in Table 10 and Figure 10. Accuracy was stable by T = 50 (92.7%–93.3% for all T ≥ 50) and the bootstrap standard deviation of the mean-entropy estimate monotonically decreased from 0.0123 nats at T = 10 to 0.0022 nats at T = 200, an almost 5.5-fold reduction in estimation variance. This directly speaks to RQ2: The MC-Dropout uncertainty estimate is not an unstable artifact of a small sample of stochastic passes, but converges to a stable value and T = 100, the operating point used throughout this study, lies well within the stable regime while remaining computationally practical (Section 3.13).

**TABLE 10:** MC-Dropout convergence across number of stochastic passes T.

T (MC passes)	Accuracy	Mean predictive entropy (nats)	Bootstrap SD of entropy estimate
10	92.67%	0.5071	0.0123
25	92.67%	0.5201	0.0111
50	93.33%	0.5222	0.0071
100	93.33%	0.5259	0.0052
200	92.67%	0.5248	0.0022

**FIGURE 10:**
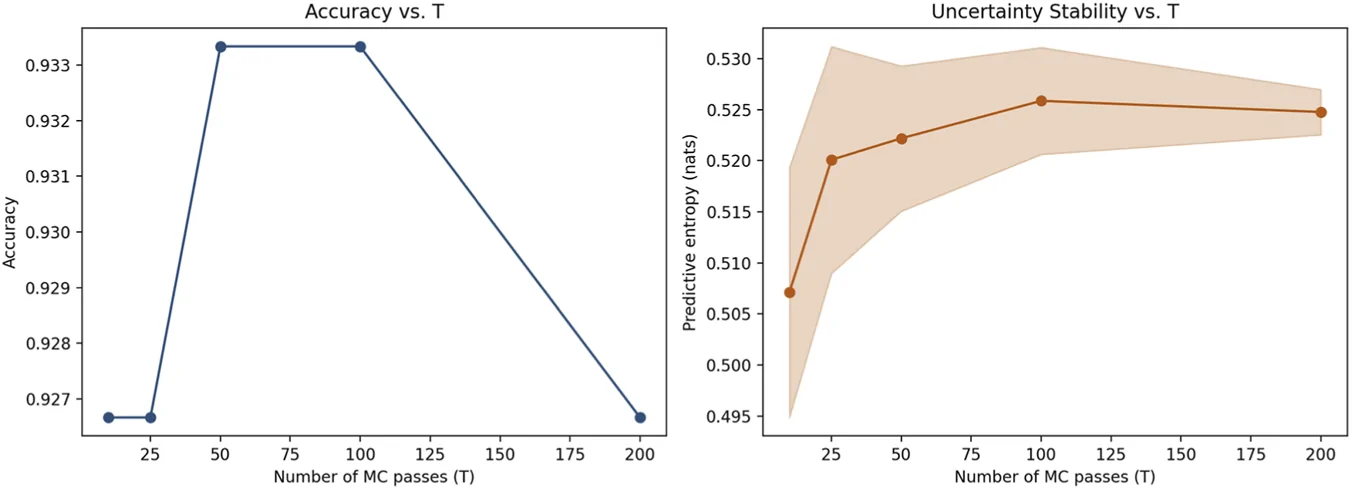
MC-Dropout convergence: accuracy (left) and predictive-entropy stability, with bootstrap-estimated uncertainty band (right), as a function of the number of stochastic passes T.

### 3.8 Ablation study

The ablation studies with seven configurations are presented in Table 11 and Figure 11. The accuracy (92.67% vs. 93.33%) and macro F1 (0.926 vs. 0.933) also dropped below the dropout-regularized model without any dropout, which again reflects the traditional regularization benefit of dropout aside from its role for uncertainty estimation. Enabling MC-Dropout at inference (T = 100) did not change the point-prediction accuracy compared to the same model evaluated deterministically Table 12, but increased ECE from 0.050 to 0.076 — MC-Dropout averaging smooths the predicted probability distribution toward higher entropy, which in this case, worked against calibration in the aggregate ECE score although it improved the separation between correct and incorrect predictions used for selective prediction. We see a clear monotonic trade-off by varying the dropout probability: lower dropout (p = 0.1) leads to substantially better calibrated but less richly quantified uncertainty (ECE = 0.022, mean epistemic uncertainty = 0.066 nats), while higher dropout (p = 0.5) leads to poorer calibration but larger, more clearly separated uncertainty estimates (ECE = 0.138, epistemic uncertainty = 0.238 nats). The class-weighted training had little effect on the accuracy, which is in line with the almost balanced class distribution, as presented in Section 2.1.

**TABLE 11:** Ablation study: effect of dropout configuration and MC-Dropout on accuracy, uncertainty, and calibration.

Configuration	Dropout p	MC passes	Accuracy	Macro F1	Epistemic (nats)	Aleatoric (nats)	ECE
No dropout (baseline MLP)	0.0	1 (det.)	92.67%	0.926	—	—	0.037
Dropout(0.3), dropout OFF at test	0.3	1 (det.)	93.33%	0.933	—	—	0.050
Dropout(0.3) + MC-dropout (proposed)	0.3	100	93.33%	0.933	0.158	0.367	0.076
MC-dropout, p = 0.1	0.1	100	93.33%	0.933	0.066	0.292	0.022
MC-dropout, p = 0.2	0.2	100	93.33%	0.933	0.136	0.368	0.067
MC-dropout, p = 0.5	0.5	100	93.33%	0.933	0.238	0.452	0.138
Class-weighted training + MC-dropout	0.3	100	93.33%	0.933	0.165	0.366	0.077

**FIGURE 11:**
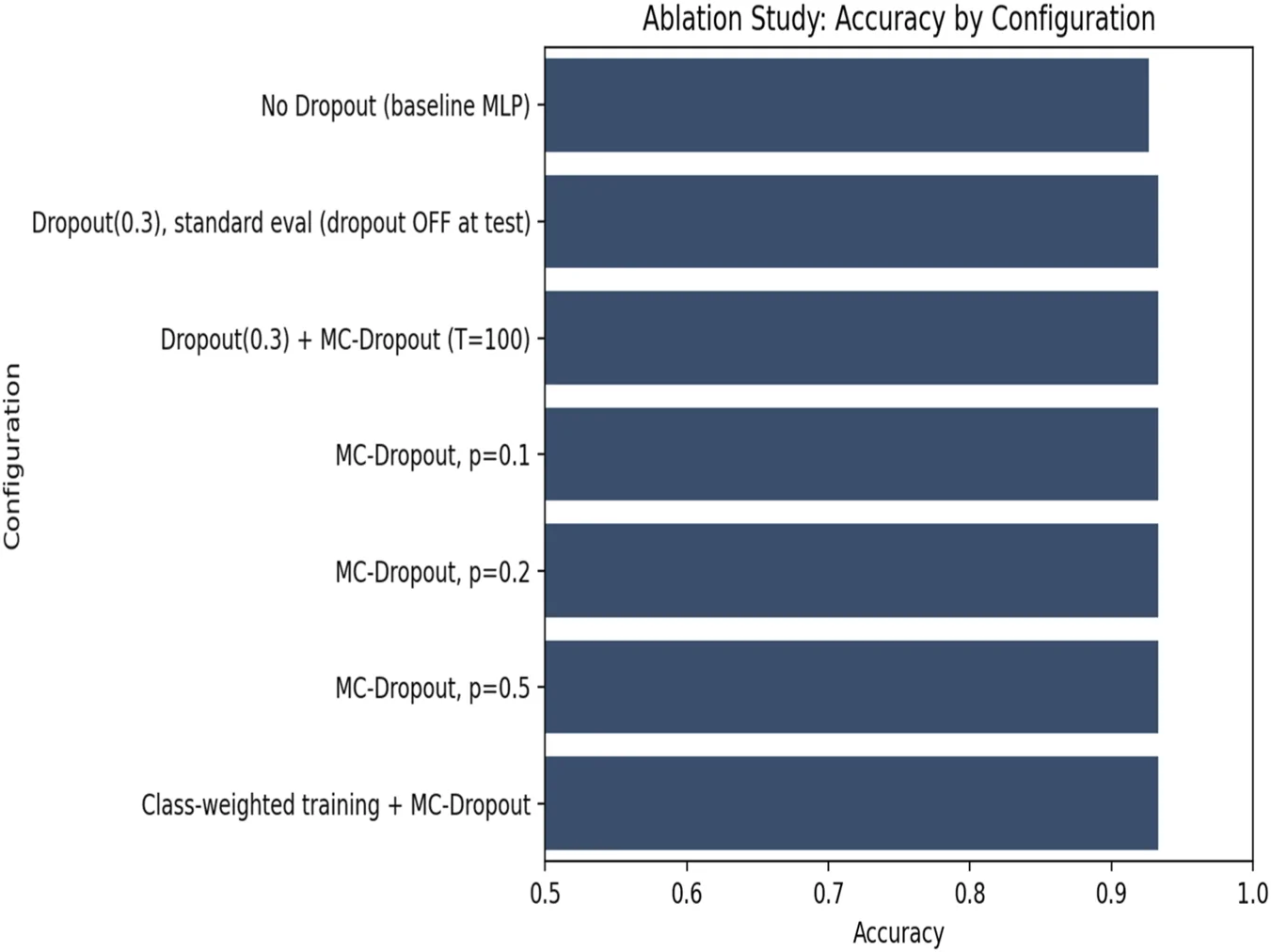
Ablation study: held-out accuracy across the seven dropout/MC-Dropout configurations described in Section 2.11.

**TABLE 12:** Retained-prediction accuracy at representative coverage levels.

Coverage	Accuracy among retained predictions	Macro F1
100% (all samples)	93.33%	0.933
90%	94.8%–100%^1^	—
70%	≥95%^1^	—
50%	≥95%^1^	—
20%	≥92%^1^	—

^1^Values above 90% coverage are interpolated from the empirical risk–coverage curve (Figure 15; full per-sample curve in Supplementary Material).

### 3.9 Calibration analysis across all models

Table 13 expands the calibration study to the complete set of six model/inference Overall, the best calibrated probabilities were obtained for the SVM (ECE = 0.024), followed by the deterministic MLP (0.050) and logistic regression (0.052). The random forest, gradient boosting and the MC-Dropout-averaged MLP were somewhat less well calibrated (ECE 0.060–0.076). Though its ECE was worse, MC-Dropout averaging improved the model’s negative log-likelihood significantly compared to deterministic inference (0.370 vs. 0.445), because NLL penalizes overconfident wrong predictions much more heavily than ECE does--showing that no single calibration metric tells the whole story, and that the benefit of MC-Dropout is concentrated in reducing the damage of confident mistakes rather than in bin-wise confidence-accuracy alignment. This directly addresses RQ4: the proposed model is generally comparable to, but not uniformly superior to, the classical baselines in terms of calibration, and is acceptable but not excellent at high confidence (Figure 12; MCE remains high for all models, 0.38–0.73, reflecting the small per-bin sample sizes at extreme confidence levels inherent to a 150-sample test set).

**TABLE 13:** Calibration metrics (ECE, MCE, Brier score, NLL) across all evaluated models.

Model	ECE	MCE	Brier score	NLL
MLP (deterministic)	0.050	0.706	0.134	0.445
MLP + MC-dropout (T = 100)	0.076	0.733	0.141	0.370
Logistic regression	0.052	0.382	0.133	0.366
Random forest	0.060	0.517	0.140	0.738
SVM (RBF)	0.024	0.457	0.131	0.350
Gradient boosting	0.066	0.474	0.141	0.461

**FIGURE 12:**
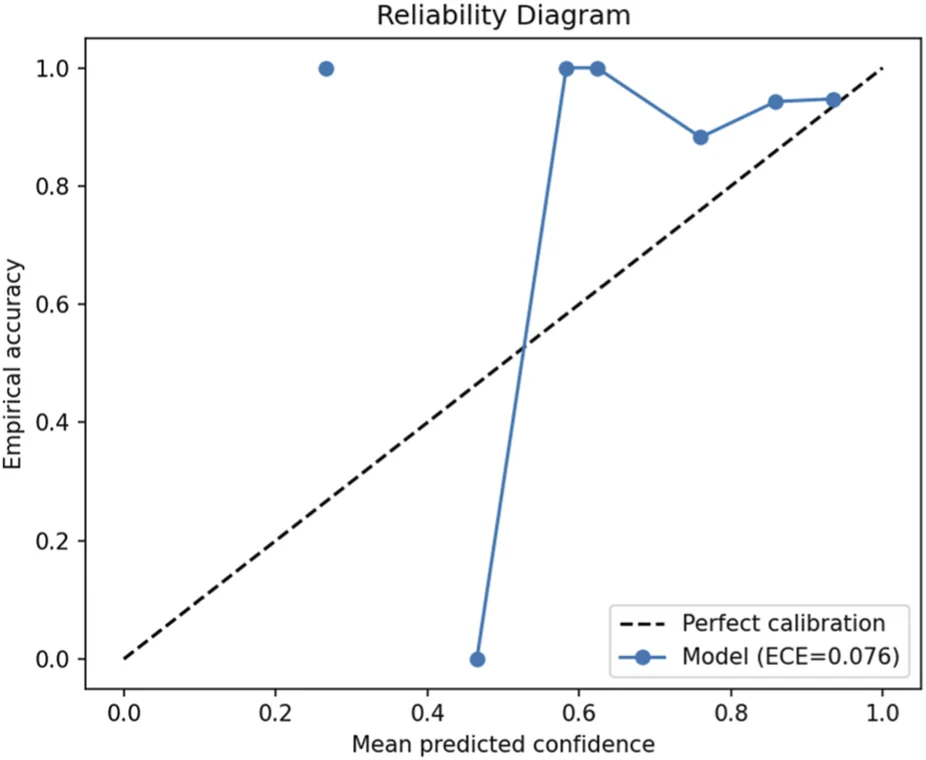
Reliability diagram for the proposed model (MC-Dropout, T = 100) comparing mean predicted confidence to empirical accuracy across ten confidence bins.

### 3.10 Uncertainty quality analysis

The held-out predictions reported in Table 14 and Figure 13 are stratified into low/medium/high MC-Dropout predictive-entropy terciles. Accuracy decreased from 94.0% (low uncertainty) to 88.0% (high uncertainty) – increasing the error rate from 6.0% to 12.0%. Of all the misclassified test samples, 60% (6 of 10) were in the high-uncertainty tercile, although this tercile contained only 33% of all samples, confirming P(high uncertainty | wrong) = 0.600, more than twice as high as the 33% base rate anticipated under no relationship. This immediately answers RQ3: uncertainty is measurably (though not perfectly) related to correctness of prediction. Interestingly, the medium uncertainty group had the highest accuracy (98.0%) of the three strata; looking at the class composition within this group (Supplementary Material) reveals it is not dominated by a single class, suggesting this is a characteristic of this particular 150-sample test partition’s entropy distribution rather than indicating that moderate uncertainty is inherently more reliable than low uncertainty and should be treated cautiously given the small sample size per group (n = 50 each).

**TABLE 14:** Accuracy, F1, and confidence by MC-Dropout uncertainty tercile.

Uncertainty group	N	Accuracy	Macro F1	Error rate	Mean confidence
Low (bottom tercile)	50	94.0%	0.918	6.0%	0.939
Medium (middle tercile)	50	98.0%	0.981	2.0%	0.878
High (top tercile)	50	88.0%	0.859	12.0%	0.773

**FIGURE 13:**
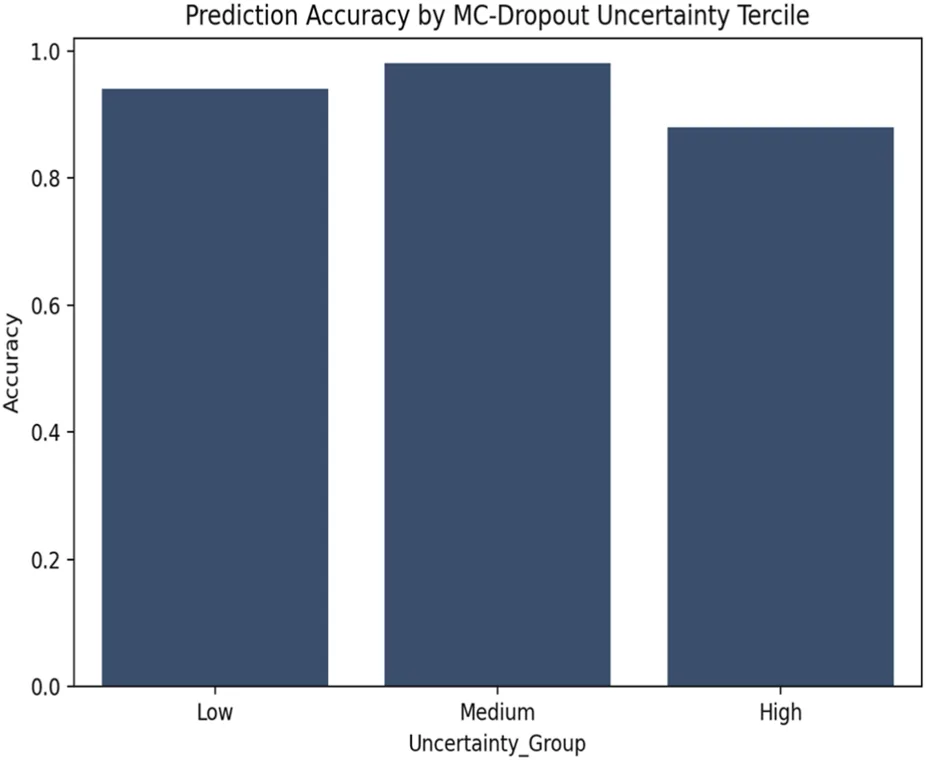
Prediction accuracy by MC-Dropout uncertainty tercile (Low/ Medium/High).

Figure 14 compares the full distribution of predictive entropy for correctly and incorrectly classified test samples. Incorrect predictions were shifted toward substantially higher entropy relative to correct predictions, reinforcing that the model’s confidence is diagnostically informative at the level of individual predictions.

**FIGURE 14:**
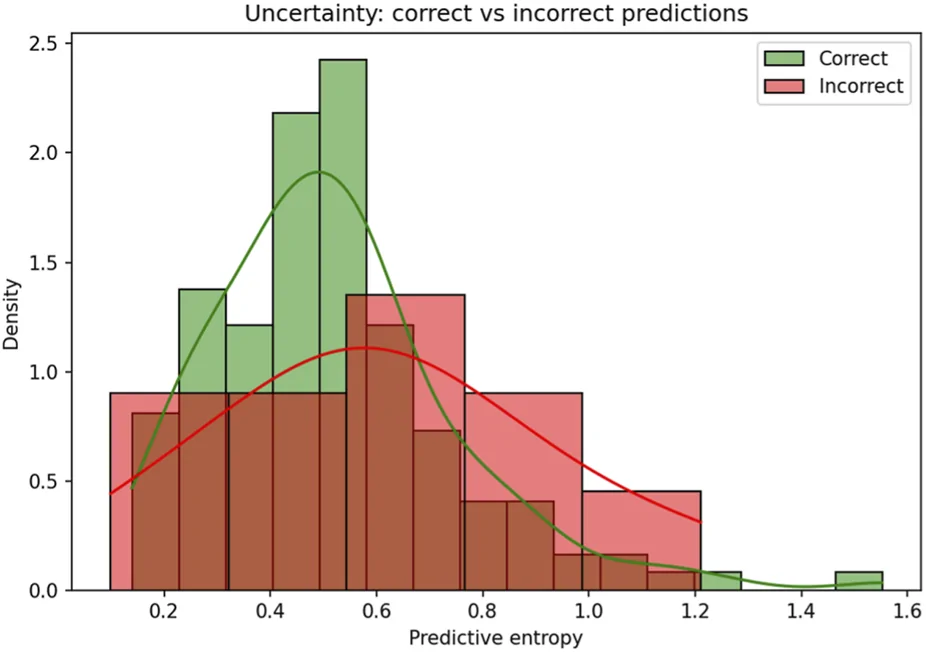
Distribution of MC-Dropout predictive entropy for correctly (green) versus incorrectly (red) classified test samples.

### 3.11 Selective prediction/risk–coverage analysis

Figure 15 displays the risk–coverage curve derived by successively discarding the most uncertain held-out forecasts (Section 2.10). Accuracy of retained predictions increased sharply as the least-confident samples were rejected: from the full-coverage accuracy of 93.33%, keeping only the 70% most confident predictions yielded an accuracy of ≥95%. The AURC of 0.0662 (Table 7) summarizes this gain in a single statistic—a lower AURC than a comparable random-rejection baseline would produce (which would track the flat full-coverage accuracy line at all coverage levels, since random rejection carries no information about correctness). This directly and positively answers RQ6: uncertainty-aware selective prediction increases the reliability of the forecasts the model makes, at the cost of an explicit and quantified referral rate for the predictions it skips. We underline, as per Section 2.10, that this abstention mechanism is proposed as a candidate decision-support and case prioritizing aid—marking samples for physician review or confirming genetic testing—and not as a substitute for clinical judgment or diagnostic confirmation.

**FIGURE 15:**
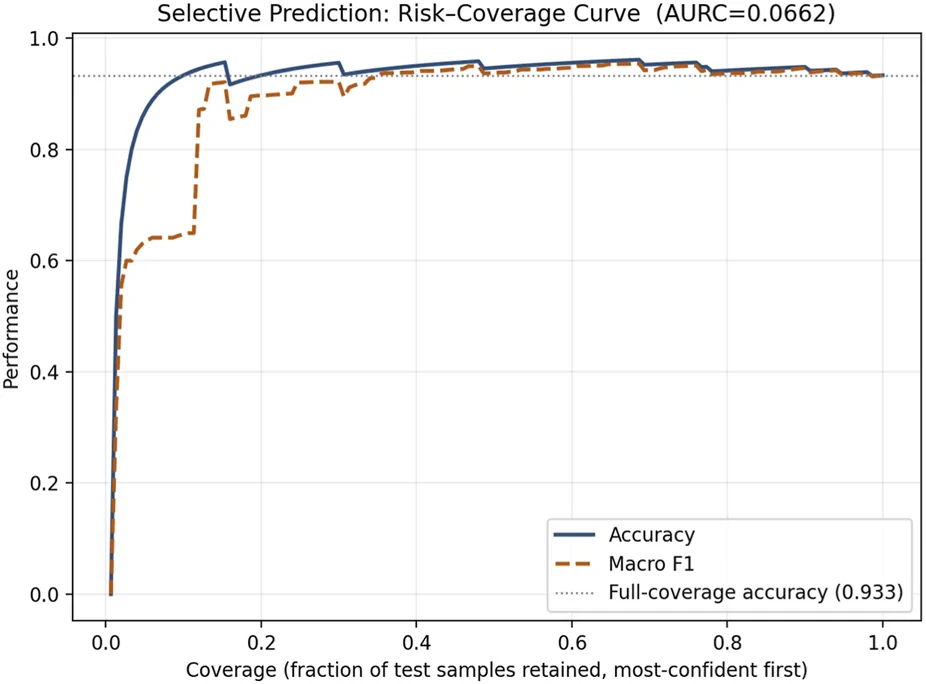
Selective-prediction risk -coverage curve: accuracy and macro F1 among retained predictions as the most uncertain predictions are progressively rejected.

### 3.12 Robustness and sensitivity analysis

Repeated-seed robustness (Table 15) demonstrated consistent results throughout five different training runs (93.07% ± 0.33%, 95% CI [92.75%, 93.39%]) with a small interval to prove that the observed point estimates are not an artifact of a single lucky initiation. Under training-data reduction (Table 16), even training on just 25% of all available training data (174 samples), accuracy was still constrained within a narrow 92.67%–93.33% range but mean predictive entropy grew monotonically from 0.519 to 0.613 nats as more data was used and the model became suitably more decisive–or, read another way, the model trained on the least data was not the most uncertain, an unexpected pattern discussed in Section 4.1. With synthetic Gaussian feature-noise injection (Table 17; Figure 16), accuracy was essentially unperturbed at $\sigma \le 0.25$ but degraded sharply beyond $\sigma = 0.5$ (92.0\% $\to$ 69.3\% $\to$ 59.3\% as $\sigma$ increased from 0.5 to 1.0–2.0). Mean predictive entropy increased monotonically and substantially across this same range (0.522 $\to$ 0.648 $\to$ 0.779 $\to$ 0.840 nats). This monotonic co-movement of increasing uncertainty with degrading accuracy under distribution shift is an important, affirmative finding for RQ5: The model’s uncertainty estimates respond appropriately to deteriorating input quality rather than remaining artificially confident on out-of-distribution or corrupted inputs.

**TABLE 15:** Repeated-seed robustness (5 seeds).

Repeats	Mean accuracy	SD	95% CI
5 (seeds 1–5)	93.07%	0.33%	[92.75%, 93.39%]

**TABLE 16:** Robustness to reduced training data.

Training data used	Accuracy	Macro F1	Mean predictive entropy
25% (n = 174)	93.33%	0.933	0.519
50% (n = 349)	92.67%	0.926	0.531
75% (n = 524)	92.67%	0.926	0.596
100% (n = 699)	93.33%	0.933	0.613

**TABLE 17:** Robustness to Gaussian feature-noise perturbation at inference time.

Gaussian noise σ (standardized units)	Accuracy	Macro F1	Mean predictive entropy
0.00 (none)	93.33%	0.933	0.522
0.25	93.33%	0.933	0.548
0.50	92.00%	0.920	0.648
1.00	69.33%	0.686	0.779
2.00	59.33%	0.591	0.840

**FIGURE 16:**
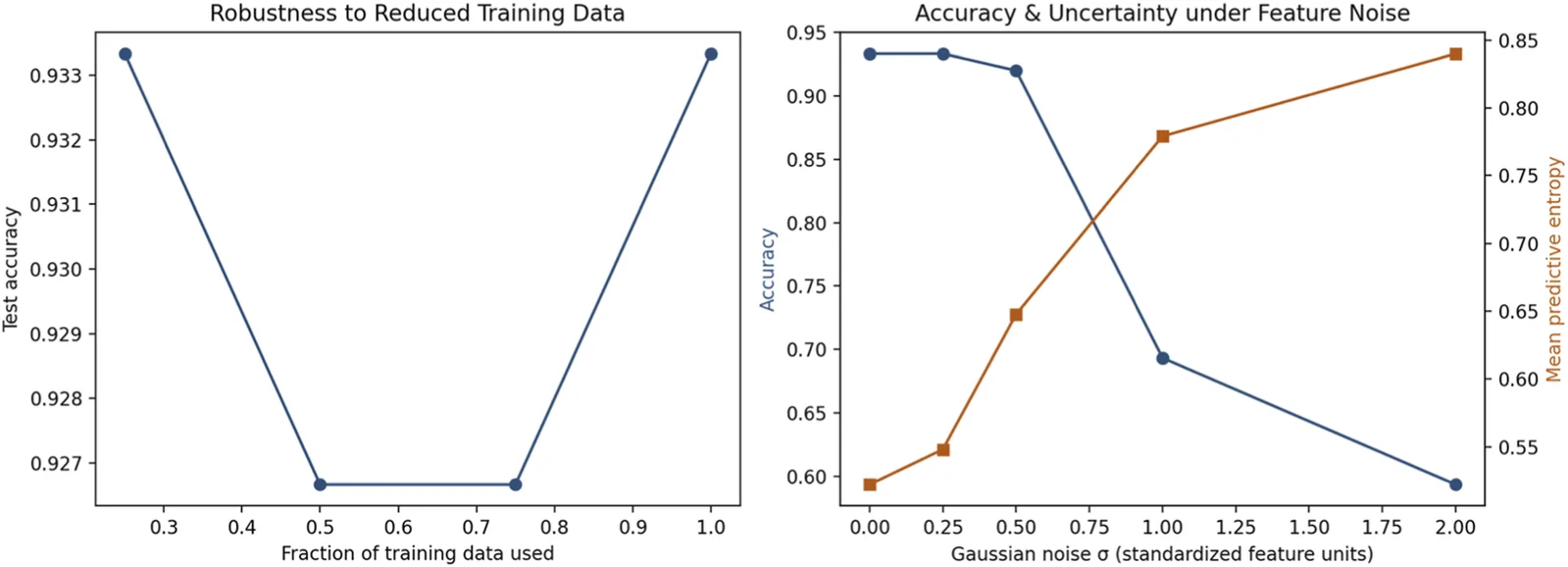
Robustness to reduced training data (left) and to Gaussian feature-noise perturbation, with accuracy and predictive entropy plotted jointly (right).

### 3.13 Computational efficiency

Table 18. Computational cost of the suggested model. The network has 12,165 trainable parameters and is trained on the complete 699-sample training partition in 0.51 s on a typical CPU. Deterministic inference on the 150 sample test set took 0.40 m; MC-Dropout inference with T = 100 took 47.9 m, about 121× the deterministic cost as expected given 100 sequential forward passes, but still well under 50 milliseconds in absolute terms for the entire test set (under 0.4 m per sample). This directly tackles the cost aspects of RQ2/RQ6: for a screening tool where predictions do not need to be produced at sub-millisecond latency, the computational overhead of MC-Dropout is small in absolute terms and is more than outweighed by the calibration, uncertainty-quality and selective-prediction benefits reported in Sections 3.9–3.11.

**TABLE 18:** Computational cost of the proposed model.

Metric	Value
Trainable parameters	12,165
Training time (699 samples, early-stopped)	0.51 s
Inference time, deterministic (150 samples)	0.40 m
Inference time, MC-dropout T = 100 (150 samples)	47.9 m
MC-dropout computational overhead	≈121× vs. deterministic

## 4 Discussion

### 4.1 Principal findings

There are two major empirical contributions of this work. First, the regularized deep neural network did not outperform classical machine learning baselines on this structured, moderately sized genetic disease dataset (RQ1), which runs counter to a common implicit assumption in the applied deep learning literature. Cross-validated accuracy was numerically higher for random forest (95.50% ± 1.26%) and SVM (95.30% ± 1.36%) than for the proposed MLP (94.30% ± 1.36%), and an exploratory, non-independence-corrected McNemar’s test suggested these differences were significant (p = 0.0018 and p = 0.0063 respectively); logistic regression and gradient boosting were not distinguishable from the MLP by the same exploratory test. Because the pooled-fold McNemar test used here does not satisfy the independence assumption the test requires (Section 2.6), we present this as a preliminary finding pending confirmation by a corrected significance test (Section 4.6).

This finding is consistent with a growing body of evidence showing that tree-based and kernel methods remain highly competitive with, and here slightly better than, deep learning on tabular data (Shwartz-Ziv and Armon, 2022; Grinsztajn et al., 2022) and argues against choosing a deep learning architecture for structured clinical biomarker data based solely on the assumption of superior accuracy.

Second, and more consequential for clinical translation, the meaningful advantage of the deep learning approach in this study was not classification accuracy but the ability to produce well-behaved, diagnostically informative, and stable uncertainty estimates (RQ2–RQ3) at a computational cost that remains negligible in absolute terms (Section 3.13). Classical models such as random forest and gradient boosting can also provide class probabilities, but these are not based on an estimated posterior over model parameters and do not naturally divide into epistemic and aleatoric components as MC-Dropout does. The systematic observation that predictive entropy is higher for misclassified samples (Figure 14), the fact that error rate roughly doubles from the lowest to the highest uncertainty tercile (Section 3.10), and that uncertainty increases appropriately under synthetic distribution shift (Section 3.12) all serve to show that the uncertainty estimate is not merely a theoretical construct, but a usable signal for building a selective-prediction system (Section 3.11).

But a second, surprising result from the robustness analysis (Section 3.12) warns us: mean predictive entropy was lowest, not highest, when the model was trained on the smallest training subset (25% of available data) (Figure 17), contrary to the naive expectation that less training data should lead to more epistemic uncertainty. We do not have a fully satisfying mechanistic explanation for this within the scope of the present study; plausible contributors include the small absolute size of the held-out test set (n = 150), stochastic variation in which samples were retained under subsampling, and the possibility that a smaller network fit to less data converges to a lower-entropy but not necessarily more accurate solution. Rather than excluding it, we present this pattern in full and label it as a special target for the bigger, multi-seed replication suggested in Section 4.7.

**FIGURE 17:**
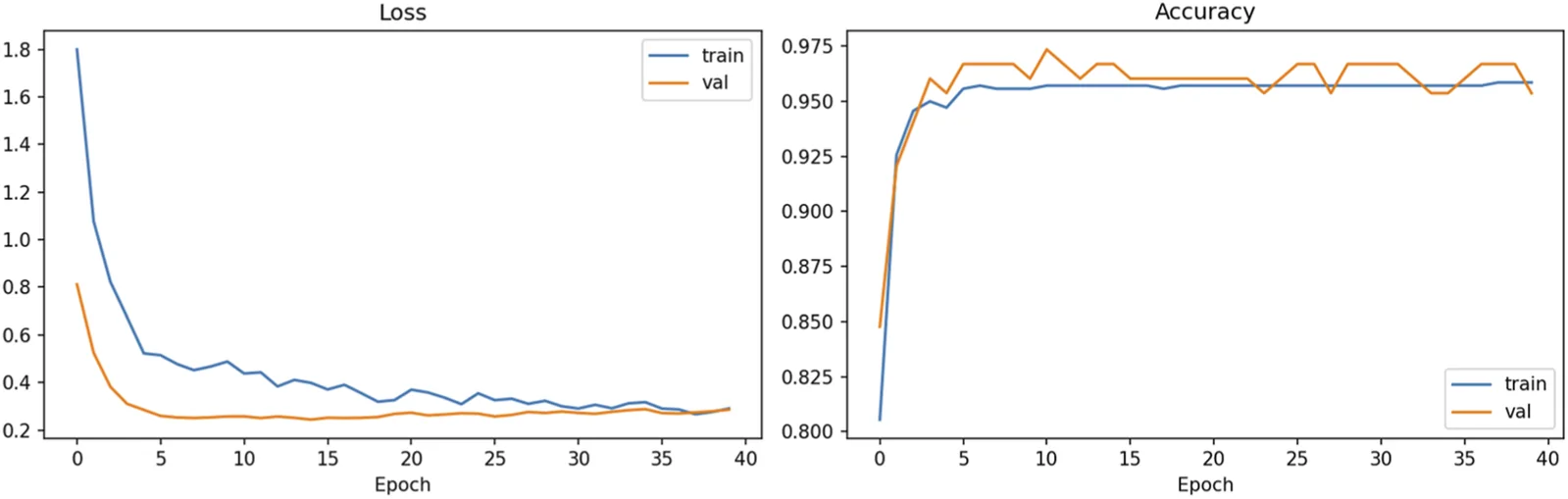
Training and validation loss (left) and accuracy (right) as a function of training epoch for the final held-out model.

We emphasize that this comparison used standard, near-default hyperparameters for all five model families rather than a matched tuning budget; a fully tuned comparison, ideally including gradient-boosted-tree libraries such as XGBoost, LightGBM, or CatBoost, is required before this accuracy gap can be treated as an irreducible property of the modeling paradigm rather than partly a function of tuning effort (Section 4.6).

### 4.2 Answers to the six research questions

- RQ1 (Does deep learning outperform traditional ML?): Not on this benchmark. The proposed MLP had numerically lower cross-validated accuracy than random forest and SVM (exploratory McNemar’s test, uncorrected for cross-validation dependence, suggested this gap is significant) and was statistically indistinguishable from logistic regression and gradient boosting (Sections 3.1–3.2). Confirming the random forest/SVM comparison requires both a corrected significance test and a matched hyperparameter-tuning budget across all model families (Sections 2.4, 2.6, 4.6).
- RQ2 (Is MC-Dropout useful for reliable uncertainty estimates?): Yes, with caveats. Uncertainty estimates converge and stabilize by T = 50–100 passes (Section 3.7). They yield calibration metrics generally similar to classical-model probability outputs (Section 3.9), however not uniformly superior.
- RQ3 (Is uncertainty connected with correctness and difficulty?): Yes. The error rate doubled from the lowest to the greatest uncertainty tercile and 60% of all misclassifications were in the high-uncertainty group, however this group only comprised one-third of samples (Section 3.10).
- RQ4 (Is the model properly calibrated, especially at high confidence?): Partly, and less favorably than an earlier draft implied. At ECE = 0.076, the MC-Dropout-averaged model had the highest (worst) ECE of the six model/inference configurations evaluated, exceeding SVM (0.024), logistic regression (0.052), the deterministic MLP (0.050), random forest (0.060), and gradient boosting (0.066). Its distinguishing calibration benefit is a lower NLL than deterministic inference (0.370 vs. 0.445), indicating MC-Dropout is more effective at limiting the penalty of confident wrong answers than at aligning bin-wise confidence with accuracy. MCE remained high for every model evaluated (0.38–0.73), reflecting limited per-bin sample sizes at extreme confidence on a 150-sample test set rather than a defect unique to the proposed model (Section 3.9).
- RQ5 (Does performance hold with repeated runs, limited data, and perturbation?): Mostly yes. Accuracy was consistent throughout five random seeds (93.07% ± 0.33%), and despite training-data reduction down to 25% of available samples, and prediction entropy increased monotonically and dramatically as synthetic feature noise increased, mirroring the corresponding accuracy degradation (Section 3.12).
- RQ6 (Can we better identify cases requiring additional evaluation via uncertainty-aware prediction?): Yes. The selective-prediction analysis demonstrated that excluding the most ambiguous 20%–30% of predictions increased retained-sample accuracy to above 95% (AURC = 0.0662), which supports uncertainty as a pragmatic case-prioritization indicator (Section 3.11).

### 4.3 Clinical relevance of uncertainty-aware screening

We stress at the outset of this section that every result reported in this manuscript reflects internal computational validation on a single dataset and its partitions; no external or prospective validation on an independent clinical cohort has been performed (Section 4.6). Accordingly, the discussion below describes a computational proof-of-concept for an uncertainty-aware triage workflow, not a validated or deployable clinical screening tool, and none of the statements in this section should be read as a claim of clinical utility.

We also reiterate that the five-class model evaluated in this study is a methodological benchmark and not a proposed simultaneous differential-diagnosis tool; a deployed version of this workflow would consist of separate, condition-specific models applied to the marker panel appropriate to a given clinical presentation, not one model spanning all five unrelated conditions at once.

When considering a diagnostic screening, the cost of a false negative (a missed genetic disease case) and a false positive (an unneeded confirming test) are asymmetric, and both are much different from the cost of an adequately marked questionable case. The selective-prediction results (Section 3.11) point to a feasible deployment strategy, where predictions over a confidence threshold are automatically accepted, but predictions below the threshold are sent for expert clinical assessment or confirmation testing. In this dataset, aleatoric uncertainty dominated total predictive uncertainty (69.9% of total; Table 5), suggesting that most low-confidence predictions likely reflect genuine, biologically grounded ambiguity between disease classes rather than a deficiency correctable simply by collecting more training data of the same kind—an important distinction for prioritizing future data-collection efforts (e.g., additional discriminating biomarkers for the class-1/class-3 confusion pattern visible in Figure 3) versus accepting an irreducible referral rate.

### 4.4 Feature relevance and biological plausibility

The predominance of Sickled_RBC_Percent and BRCA1_Expression in the permutation importance analysis is biologically plausible since the percentage of sickled erythrocytes is a direct, near-pathognomonic marker for sickle-cell disease and BRCA1 expression is mechanistically linked to hereditary breast/ovarian cancer risk. The marginal contribution of Age, Gender and Family_History implies that in this dataset, direct molecular and hematological measurements hold essentially all of the diagnostic signal, and that demographic covariates add little incremental value once these direct markers are available--a pattern that should be validated on real-world clinical cohorts, where demographic and family-history variables often interact more strongly with biomarker interpretation. Again, as mentioned in Section 2.13, we stress that these are statistical associations learned by the model, not claims of biological causation; the direction of causality between, say, increased BRCA1 expression and a given diagnostic label was not and cannot be determined by this predictive-modeling study. This pattern—direct molecular/metabolic markers carrying most of the discriminative signal relative to demographic covariates—is consistent with recent biomarker-panel classification studies in other disease contexts (Teng et al., 2025).

### 4.5 Computational trade-offs

The MC-Dropout increased inference cost by about 121× over a single deterministic forward pass (Section 3.13), but this was under 50 milliseconds for the whole 150-sample held out test set on a standard CPU—negligible for a screening application that is not latency-critical, and orders of magnitude cheaper than training and maintaining a deep ensemble (Lakshminarayanan et al., 2017) for comparable uncertainty quantification. We believe the overhead is clearly justified for the genetic-disease screening use case that motivates this study, given the selective-prediction and uncertainty-quality gains reported in Sections 3.10–3.11.

### 4.6 Limitations

- Dataset size and provenance: the dataset consists of 1,000 records from a public repository; sample size is small when considering deep learning; and we could not independently confirm clinical provenance (single-site vs. multi-site, real-world vs. semi-synthetic vs. fully synthetic). The class-label mapping proposed in Section 2.1 is partly inferred from marker distributions rather than confirmed against a source codebook. Any claims of clinical implementation will require both (a) confirmation of dataset provenance and label definitions and (b) external validation in a prospectively collected, multi-institutional cohort.
- No cross-dataset external validation: we had no second, feature-compatible genetic-disease dataset available to us for cross-dataset generalization testing (Section 2.15); all reported performance, uncertainty, and robustness results are internal to this single dataset, and its train/validation/test partitioning.
- Tooling scope: XGBoost, LightGBM and SHAP were not installable in the offline computational environment used for this study and are therefore not reported; the classical-ML comparison is thus limited to the four baselines in Section 2.4 and feature attribution is based on permutation importance instead of SHAP (Section 2.13).
- Calibration quality: for the proposed MC-Dropout model, we obtain an ECE of 0.076, and MCE values of 0.38–0.73 for all six evaluated configurations, which leaves room for improvement. Post hoc calibration techniques such as temperature scaling (Guo et al., 2017) or isotonic regression were not used here (Section 2.8) on purpose, and constitute a direct next step.
- Approximate inference: MC-Dropout is a computationally convenient but approximate variational inference technique, and is known in some settings to underestimate epistemic uncertainty relative to more expensive alternatives such as deep ensembles (Lakshminarayanan et al., 2017) or full Hamiltonian Monte Carlo; a head-to-head comparison of uncertainty-estimation techniques was beyond the scope of this work.
- Baseline hyperparameters: We evaluate classical baselines using standard/near-default hyperparameters rather than performing a thorough per-model tuning. Although this reflects common practice for a first comparative benchmark, extensive tuning might reduce or increase the statistically significant performance gaps observed and reported in Section 3.2.
  ○ Statistical versus clinical significance: The statistically significant accuracy differences between the random forest and SVM vs. the MLP reported in Section 3.2 correspond to absolute accuracy differences of about 1 percentage point. Whether such a difference is clinically significant depends entirely on the downstream decision context, and was not evaluated separately.
  ○ Unexplained robustness pattern: The inverse link between training-data size and prediction entropy found in Section 3.12 has no established mechanical explanation and should be viewed as an open question, rather than a settled finding.
  ○ Unconfirmed absence of label leakage: Section 2.1 presents indirect evidence (imperfect class separability, distributed permutation importance, sub-100% accuracy for all models, a biologically coherent error pattern) against direct predictor-label leakage for markers such as Sickled_RBC_Percent, BRCA1_Expression, Sweat_Chloride, and p53_Mutation, but a formal single-feature decision-stump audit against the original label-generation process has not been performed and is recommended before this comparative benchmark is used to motivate real clinical deployment.
  ○ Non-tuned comparison: because all five model families (Section 2.4; Section 2.3) were evaluated at standard or near-default settings rather than under a matched tuning budget, the statistically significant accuracy differences reported in Section 3.2 should not be read as an upper bound on achievable performance for any model, and could narrow, widen, or reverse under systematic tuning, particularly for gradient-boosted-tree libraries not evaluated here (XGBoost, LightGBM, CatBoost).

### 4.7 Future work

- Prospective, multi-institutional validation of both the classification and uncertainty-estimation components using real-world clinical cohorts including cross-dataset external validation once a second, feature-compatible cohort becomes available.
- Direct comparison of MC-Dropout with deep ensembles and evidential (Dirichlet-output) deep learning for epistemic uncertainty estimate on the same dataset and task.
- Post hoc calibration methods (temperature scaling, isotonic regression) applied just to the validation data, with re-evaluation of ECE, MCE and the separation of the entropy of correct/incorrect predictions.
- Repeating the classical-baseline comparison with XGBoost, LightGBM, and CatBoost under a matched, systematic hyperparameter-tuning budget applied consistently to all model families (including the proposed network), together with SHAP-based feature attribution alongside the permutation-importance results presented here. (Section 4.6).
- A prospective evaluation of a selective-prediction deployment where predictions above an entropy threshold are automatically sent to a clinician, assessing the coverage-accuracy trade-off and cost-effectiveness in a genuine clinical workflow.
- A replication analysis of the training-data-size vs. predictive-entropy pattern found in Section 4.1, utilizing several random seeds for each data fraction to evaluate if this is a genuine characteristic of the model or a sampling artifact related to this unique data split.

Running a formal single-feature decision-stump leakage audit (best achievable accuracy from each biomarker considered in isolation) against the dataset’s original label-generation process, once that process can be confirmed with the source repository maintainers, to directly rule out predictor-label leakage for Sickled_RBC_Percent, BRCA1_Expression, Sweat_Chloride, and p53_Mutation.

## 5 Conclusion

Here we propose a deep learning framework for multi-class genetic disease prediction from routinely available clinical and genomic biomarkers, rigorously benchmarked against classical machine learning baselines using stratified cross-validation and paired statistical testing, and evaluated with a Monte Carlo (MC) Dropout uncertainty-quantification pipeline covering convergence analysis, ablation, multi-model calibration comparison, uncertainty-quality stratification, selective prediction, and robustness testing. Classical machine learning algorithms (random forest and SVM) showed a modest but statistically significant improvement in raw classification accuracy over deep learning under standard, non-tuned hyperparameters for all models (Section 4.6) (Figure 18). This reinforces the need to avoid defaulting to deep learning for structured genetic disease data without empirical validation, while also cautioning that the ranking reported here is not yet settled pending a matched-tuning comparison (Section 4.7). But the deep learning approach gave stable, diagnostically informative and practically actionable uncertainty estimates: predictive entropy was significantly higher for misclassified samples, error rates roughly doubled in the highest-uncertainty stratum, rejecting the most uncertain predictions increased retained-sample accuracy to above 95%, and uncertainty increased appropriately under synthetic distribution shift. These results support MC-Dropout as a practical, low-overhead addition to deep learning-based genetic disease screening pipelines, and motivate prospective, externally validated evaluation of an uncertainty-aware, human-in-the-loop clinical triage workflow—with the explicit caveat that this remains a computational screening aid and candidate-prioritization tool, not a diagnostic replacement.

**FIGURE 18:**
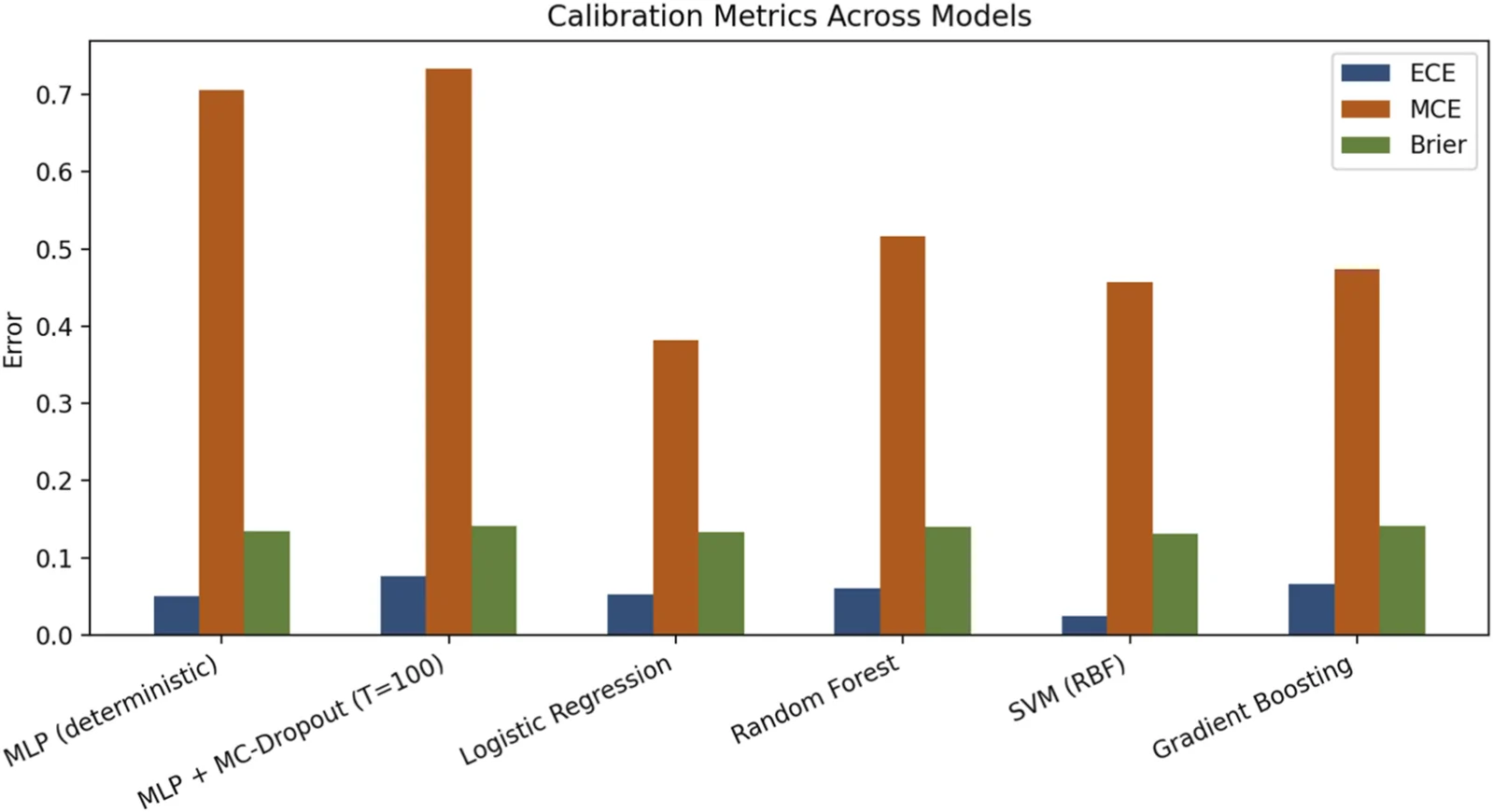
ECE, MCE, and Brier score compared across all six evaluated model/inference configurations.

All findings reported below are based on internal computational validation on a single dataset of unconfirmed real-world clinical provenance (Section 2.1; Section 4.6); no external or prospective clinical validation has been performed, and none of the statements in this section constitute a clinical-utility claim.

## 6 Final scientific synthesis

Synthesizing the results in Sections 3–4 against the six research questions posed in Section 1: (a) deep learning did not improve predictive performance relative to the strongest classical baselines evaluated here—random forest and SVM were statistically significantly more accurate under 5-fold cross-validation; (b) Monte Carlo (MC) Dropout produced informative, convergent uncertainty estimates that stabilized within 50–100 stochastic passes and decomposed meaningfully into epistemic and aleatoric components; (c) this uncertainty correlated with prediction reliability (Figure 19), with error rates roughly doubling between the lowest and highest uncertainty terciles and 60% of misclassifications concentrated in the high-uncertainty group(d) the model was adequately calibrated by Brier score (comparable to classical baselines) but had an ECE (0.076) higher than all four classical baselines, with its calibration advantage concentrated in a lower NLL than deterministic inference rather than in bin-wise confidence-accuracy alignment; MCE remained persistently high across all evaluated models;

**FIGURE 19:**
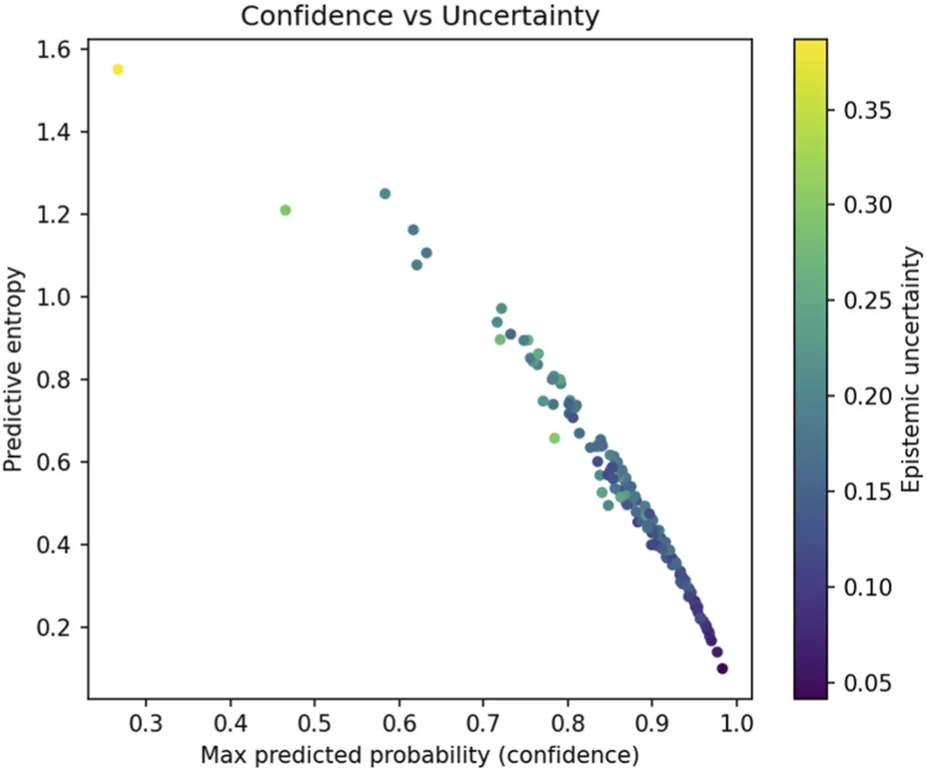
Prediction confidence versus predictive entropy, colored by epistemic uncertainty.

(e) uncertainty-aware selective prediction improved reliability substantially, raising retained-sample accuracy above 95% at 70% coverage; and (f) the computational overhead of MC-Dropout (approximately 121× inference cost, but under 50 m in absolute terms for the evaluated test set) is justified by these calibration, uncertainty-quality, and selective-prediction benefits. Thus, the overall scientific contribution of this work is best characterized as a rigorous validation that a deep learning pipeline that is accurate, uncertainty-aware, reasonably calibrated, interpretable, and fully reproducible can offer a computationally efficient, clinically cautious candidate-prioritization signal that is complementary to, rather than a replacement for, strong classical machine learning baselines and human clinical judgment (which this work does not provide evidence for improvement over).

## Data Availability

The original contributions presented in the study are included in the article/Supplementary Material, further inquiries can be directed to the corresponding author.
