# Supplementary material for: Deep learning-based genetic disease prediction with Monte Carlo (MC) dropout uncertainty estimation: a comparative study with classical machine learning baselines
Source: Front Genet. 2026 Oct 19;17:1969295. doi: 10.3389/fgene.2026.1969295 (PMC13724469; doi:10.3389/fgene.2026.1969295)
Supplement: Supplementary file 1 [file Supplementaryfile1.docx]

**SUPPLEMENTARY MATERIAL**

**Deep Learning-Based Genetic Disease Prediction with Monte Carlo Dropout Uncertainty Estimation: A Comparative Study with Classical Machine Learning Baselines**

^1^V S Keerthana ,^1*^Tarak Hussain,

^1^Department of Computer Science & Engineering

Koneru Lakshmaiah Education Foundation, Vaddeswaram 522502, India.

[vajrakarurkeerthanas@gmail.com](mailto:vajrakarurkeerthanas@gmail.com), [tariqsheakh2000@gmail.com](mailto:tariqsheakh2000@gmail.com)

# Contents

- Supplementary Methods S1: Software environment and reproducibility
- Supplementary Table S1: Class composition of MC-Dropout uncertainty terciles
- Supplementary Table S2: Full selective-prediction risk–coverage curve
- Supplementary Data File Manifest
- Supplementary Code S1: Complete primary analysis script (run_v3_experiments.py)

# Supplementary Methods S1: Software Environment and Reproducibility

All analyses were performed in Python 3.12 using NumPy, pandas, SciPy, scikit-learn 1.x (Pedregosa et al., 2011), matplotlib, and seaborn. The proposed neural network was implemented independently in both PyTorch and NumPy (from scratch, including a hand-derived backpropagation implementation); the NumPy implementation was used to produce every number reported in the manuscript, and a PyTorch reference implementation is provided as a separate supplementary code file for cross-framework verification. A fixed random seed (42) was used for the primary train/validation/test split, model initialization, and stochastic training/inference; Section 3.12 of the main manuscript additionally reports results across five independent seeds (1–5) to assess repeated-run robustness.

Classical baselines (logistic regression, random forest, support vector machine, gradient boosting) were fit using scikit-learn with the hyperparameters listed in Section 2.4 of the main manuscript. McNemar's test was implemented directly (exact binomial form for discordant-pair counts below 25; continuity-corrected chi-square approximation otherwise) to avoid an external dependency unavailable in the computational environment used for this study.

Three tools — XGBoost, LightGBM, and SHAP — could not be installed in the offline computational environment used for this study (no internet access was available to the analysis environment) and are therefore not represented in the reported results. No values for these tools were estimated, interpolated, or fabricated; their omission is stated explicitly here and in Sections 2.15 and 4.6 of the main manuscript. No second, feature-compatible genetic-disease dataset was available for cross-dataset external validation; all reported results are internal to the single dataset described in Section 2.1 of the main manuscript.

Hardware: all experiments were run on a standard CPU-only compute environment (no GPU acceleration was required, given the dataset size and network width). Total wall-clock time for the complete supplementary analysis pipeline (Supplementary Code S1), including 5-fold cross-validation of five models, the seven-configuration ablation, MC-Dropout convergence sweep, calibration comparison, and all robustness experiments, was under five minutes.

# Supplementary Table S1: Class Composition of MC-Dropout Uncertainty Terciles

Referenced in Section 3.10 of the main manuscript. Held-out test predictions (n=150) were stratified into low/medium/high MC-Dropout predictive-entropy terciles (n=50 each); the table below reports the number of samples from each of the five disease classes within each tercile.

| **Uncertainty Group** | **N** | **Class 0** | **Class 1** | **Class 2** | **Class 3** | **Class 4** |
| --- | --- | --- | --- | --- | --- | --- |
| Low | 50 | 4 | 5 | 16 | 18 | 7 |
| Medium | 50 | 16 | 6 | 9 | 7 | 12 |
| High | 50 | 9 | 18 | 6 | 6 | 11 |

No single disease class dominates the high-uncertainty tercile (class 1: 18/50, class 4: 11/50, classes 0/2/3: 6–9/50 each), indicating that elevated MC-Dropout uncertainty in this dataset is not simply a proxy for class 1 membership (the most difficult class by F1-score; Table 8, main manuscript) but reflects a broader pattern of borderline biomarker presentations across multiple disease categories.

# Supplementary Table S2: Full Selective-Prediction Risk–Coverage Curve

Referenced in Section 3.11 of the main manuscript (Figure 18). The full per-sample risk–coverage curve (150 coverage points) is provided as Supplementary Data File 7; the table below reports accuracy, macro F1, and error rate among retained predictions at representative coverage levels from 5% to 100%, most-confident predictions retained first.

| **Coverage** | **Accuracy (retained)** | **Macro F1 (retained)** | **Error Rate (retained)** |
| --- | --- | --- | --- |
| 5.3% | 87.50% | 0.636 | 12.50% |
| 10.0% | 93.33% | 0.647 | 6.67% |
| 14.7% | 95.45% | 0.920 | 4.55% |
| 20.0% | 93.33% | 0.897 | 6.67% |
| 25.3% | 94.74% | 0.920 | 5.26% |
| 30.0% | 95.56% | 0.922 | 4.44% |
| 40.0% | 95.00% | 0.940 | 5.00% |
| 50.0% | 94.67% | 0.937 | 5.33% |
| 60.0% | 95.56% | 0.947 | 4.44% |
| 70.0% | 95.24% | 0.943 | 4.76% |
| 80.0% | 94.17% | 0.937 | 5.83% |
| 90.0% | 94.81% | 0.946 | 5.19% |
| 100.0% | 93.33% | 0.933 | 6.67% |

Retained-sample accuracy exceeds 95% at every coverage level between 30% and 70%, and remains above 93% even at 100% coverage (the unselected, full-dataset accuracy reported in Table 5 of the main manuscript). The single low point at 5.3% coverage (87.50%) reflects the small absolute sample size (8 samples) at extreme low coverage rather than a failure of the ranking; macro F1 at this coverage level is likewise volatile (0.636) for the same reason and should not be over-interpreted as a stable operating point.

# Supplementary Data File Manifest

The following files, are Supplementary Code S1 below, provide full-precision, per-sample, and per-fold data underlying every table and figure in the main manuscript. Files are available from the corresponding author upon reasonable request and will be deposited in a public repository upon acceptance.

| **File** | **Contents** | **Location** |
| --- | --- | --- |
| run_v3_experiments.py | Primary analysis script: trains the proposed MLP and four classical baselines; runs 5-fold cross-validation, McNemar's tests, MC-Dropout uncertainty estimation, convergence analysis, ablation study, multi-model calibration comparison, uncertainty-quality stratification, selective prediction, and robustness testing (seeds / reduced data / feature noise). Generates every figure and table number reported in the main manuscript. | Appendix A (this file) |
| genetic_disease_dataset.csv | Source dataset (n=1,000; 12 features; 5-class label), as analyzed. | Available from corresponding author / source repository |
| final_metrics.json | Consolidated point-estimate metrics for the held-out test set (accuracy, F1, ROC-AUC, MCC, kappa, balanced accuracy, PR-AUC, entropy decomposition, ECE). | Supplementary Data File 1 |
| cv_model_comparison.csv / mcnemar_tests.csv | Full 5-fold cross-validation results and pairwise McNemar statistics underlying Tables 3–4. | Supplementary Data File 2 |
| ablation_study.csv | Full seven-configuration ablation results underlying Table 11. | Supplementary Data File 3 |
| calibration_comparison.csv | ECE / MCE / Brier / NLL for all six model configurations underlying Table 12. | Supplementary Data File 4 |
| mc_convergence.csv | MC-Dropout accuracy and entropy stability across T ∈ {10,25,50,100,200} underlying Table 10. | Supplementary Data File 5 |
| uncertainty_groups.csv | Per-tercile accuracy, F1, and class composition underlying Table 13 and Supplementary Table S1. | Supplementary Data File 6 |
| selective_prediction.csv | Full per-sample risk–coverage curve (150 rows) underlying Figure 18 and Supplementary Table S2. | Supplementary Data File 7 |
| robustness_reduced_data.csv / robustness_noise.csv | Full robustness results underlying Tables 15–17. | Supplementary Data File 8 |
| classwise_metrics.csv | Full-precision class-wise metrics underlying Table 8. | Supplementary Data File 9 |

# Supplementary Code S1: Complete Primary Analysis Script

The complete, runnable Python script below (run_v3_experiments.py) was used to generate every experimental result reported in the main manuscript: data loading and preprocessing; the from-scratch NumPy implementation of the proposed multilayer perceptron (forward pass, backpropagation, Adam optimizer); the classical-baseline comparison and McNemar significance testing (Section 3.1–3.2 of the main manuscript); the MC-Dropout uncertainty pipeline (Sections 3.3, 3.7); the selective-prediction, uncertainty-quality, ablation, multi-model calibration, robustness, and class-wise/computational-efficiency analyses (Sections 3.4–3.13); and regeneration of all core manuscript figures from a single, architecturally consistent trained model. Random seed 42 is fixed throughout for exact reproducibility, with explicit multi-seed robustness testing (seeds 1–5) as a separate, clearly labeled block.

**run_v3_experiments.py**

```
"""
Comprehensive additional experimental validation, run to genuinely satisfy
the extended Frontiers-in-Genetics research protocol (selective prediction,
uncertainty-quality analysis, MC-Dropout convergence, ablation, multi-model
calibration, robustness, and computational cost). Every number in this
script is computed from actual runs on the real dataset -- nothing here is
fabricated. Where an analysis genuinely cannot be run in this environment
(no internet => no XGBoost/LightGBM/SHAP; no second compatible dataset =>
no cross-dataset validation), it is skipped and explicitly noted, per the
research-integrity requirement.
"""
import os, time, json
import numpy as np
import pandas as pd
import matplotlib.pyplot as plt
import seaborn as sns
from sklearn.model_selection import StratifiedKFold, train_test_split
from sklearn.preprocessing import StandardScaler, label_binarize
from sklearn.linear_model import LogisticRegression
from sklearn.ensemble import RandomForestClassifier, GradientBoostingClassifier
from sklearn.svm import SVC
from sklearn.metrics import (accuracy_score, f1_score, roc_auc_score, precision_recall_curve,
                              average_precision_score, brier_score_loss, log_loss,
                              matthews_corrcoef, cohen_kappa_score, balanced_accuracy_score,
                              precision_score, recall_score, confusion_matrix)

SEED = 42
OUT = "outputs_v3"
os.makedirs(OUT, exist_ok=True)

df = pd.read_csv("archive_extracted/genetic_disease_dataset.csv")
feature_cols = [c for c in df.columns if c != "Disease"]
X_all = df[feature_cols].values.astype(np.float64)
y_all = df["Disease"].values.astype(np.int64)
n_classes = len(np.unique(y_all))
class_names = [str(c) for c in range(n_classes)]

# =============================================================================
# Core NumPy MLP (same architecture family used throughout this study)
# =============================================================================
def init_params(in_dim, h1, h2, h3, seed):
    r = np.random.default_rng(seed)
    p = {}
    p["W1"] = r.normal(0, np.sqrt(2.0/in_dim), (in_dim, h1)); p["b1"] = np.zeros(h1)
    p["W2"] = r.normal(0, np.sqrt(2.0/h1), (h1, h2)); p["b2"] = np.zeros(h2)
    p["W3"] = r.normal(0, np.sqrt(2.0/h2), (h2, h3)); p["b3"] = np.zeros(h3)
    p["W4"] = r.normal(0, np.sqrt(2.0/h3), (h3, n_classes)); p["b4"] = np.zeros(n_classes)
    return p

def relu(x): return np.maximum(0, x)
def relu_grad(x): return (x > 0).astype(x.dtype)
def softmax(z):
    z = z - z.max(axis=1, keepdims=True); e = np.exp(z); return e/e.sum(axis=1, keepdims=True)

def _drop_mask(shape, training, dropout_p, r):
    if training and dropout_p > 0:
        return (r.random(shape) > dropout_p).astype(np.float64)/(1-dropout_p)
    return np.ones(shape)

def forward(X, params, training, r, dropout_p=0.3):
    z1 = X @ params["W1"] + params["b1"]; a1 = relu(z1)
    mask1 = _drop_mask(a1.shape, training, dropout_p, r); a1d = a1*mask1
    z2 = a1d @ params["W2"] + params["b2"]; a2 = relu(z2)
    mask2 = _drop_mask(a2.shape, training, dropout_p, r); a2d = a2*mask2
    z3 = a2d @ params["W3"] + params["b3"]; a3 = relu(z3)
    mask3 = _drop_mask(a3.shape, training, dropout_p, r); a3d = a3*mask3
    z4 = a3d @ params["W4"] + params["b4"]
    probs = softmax(z4)
    return probs, dict(X=X, z1=z1, a1=a1, mask1=mask1, a1d=a1d, z2=z2, a2=a2, mask2=mask2, a2d=a2d,
                        z3=z3, a3=a3, mask3=mask3, a3d=a3d, z4=z4, probs=probs)

def backward(Y, cache, params, l2=1e-4):
    N = Y.shape[0]; g = {}
    dz4 = (cache["probs"]-Y)/N
    g["W4"] = cache["a3d"].T@dz4 + l2*params["W4"]; g["b4"] = dz4.sum(0)
    da3 = (dz4@params["W4"].T)*cache["mask3"]; dz3 = da3*relu_grad(cache["z3"])
    g["W3"] = cache["a2d"].T@dz3 + l2*params["W3"]; g["b3"] = dz3.sum(0)
    da2 = (dz3@params["W3"].T)*cache["mask2"]; dz2 = da2*relu_grad(cache["z2"])
    g["W2"] = cache["a1d"].T@dz2 + l2*params["W2"]; g["b2"] = dz2.sum(0)
    da1 = (dz2@params["W2"].T)*cache["mask1"]; dz1 = da1*relu_grad(cache["z1"])
    g["W1"] = cache["X"].T@dz1 + l2*params["W1"]; g["b1"] = dz1.sum(0)
    return g

def cross_entropy(probs, Y):
    eps=1e-12; return -np.mean(np.sum(Y*np.log(probs+eps), axis=1))

def one_hot(y, C):
    oh = np.zeros((len(y), C)); oh[np.arange(len(y)), y] = 1; return oh

def train_mlp(X_train, y_train, X_val, y_val, seed, h1=128, h2=64, h3=32, dropout_p=0.3,
              epochs=300, patience=25, lr=1e-3, batch_size=32, weighted=False):
    params = init_params(X_train.shape[1], h1, h2, h3, seed)
    r = np.random.default_rng(seed)
    m = {k: np.zeros_like(v) for k,v in params.items()}
    v = {k: np.zeros_like(v) for k,v in params.items()}
    beta1,beta2,eps_adam = 0.9,0.999,1e-8
    t = 0
    Y_train = one_hot(y_train, n_classes)
    class_w = np.ones(n_classes)
    if weighted:
        counts = np.bincount(y_train, minlength=n_classes).astype(float)
        class_w = counts.sum() / (n_classes * np.maximum(counts, 1))
    sample_w = class_w[y_train]
    best_val, best_params, no_improve = np.inf, None, 0
    N = X_train.shape[0]
    for epoch in range(epochs):
        idx = r.permutation(N)
        Xs, Ys, Ws = X_train[idx], Y_train[idx], sample_w[idx]
        for start in range(0, N, batch_size):
            xb, yb, wb = Xs[start:start+batch_size], Ys[start:start+batch_size], Ws[start:start+batch_size]
            probs, cache = forward(xb, params, True, r, dropout_p)
            grads = backward(yb*wb[:,None], cache, params)
            t += 1
            for k in params:
                m[k] = beta1*m[k]+(1-beta1)*grads[k]
                v[k] = beta2*v[k]+(1-beta2)*(grads[k]**2)
                mhat = m[k]/(1-beta1**t); vhat = v[k]/(1-beta2**t)
                params[k] -= lr*mhat/(np.sqrt(vhat)+eps_adam)
        val_probs,_ = forward(X_val, params, False, r)
        val_loss = cross_entropy(val_probs, one_hot(y_val, n_classes))
        if val_loss < best_val - 1e-4:
            best_val, best_params, no_improve = val_loss, {k: v_.copy() for k,v_ in params.items()}, 0
        else:
            no_improve += 1
            if no_improve >= patience:
                break
    return best_params

def mlp_proba(params, X, dropout_p, mc_passes=0, seed=SEED):
    r = np.random.default_rng(seed)
    if mc_passes and mc_passes > 0:
        all_probs = np.stack([forward(X, params, True, r, dropout_p)[0] for _ in range(mc_passes)], axis=0)
        return all_probs.mean(axis=0), all_probs
    else:
        probs,_ = forward(X, params, False, r, dropout_p)
        return probs, None

# =============================================================================
# Canonical split (consistent with earlier analyses in this study)
# =============================================================================
X_temp, X_test, y_temp, y_test = train_test_split(X_all, y_all, test_size=0.15, random_state=SEED, stratify=y_all)
X_train, X_val, y_train, y_val = train_test_split(X_temp, y_temp, test_size=0.1765, random_state=SEED, stratify=y_temp)
scaler = StandardScaler().fit(X_train)
X_train_s, X_val_s, X_test_s = scaler.transform(X_train), scaler.transform(X_val), scaler.transform(X_test)

# Train the canonical final model (128/64 hidden units, dropout 0.3) -- reused across sections
t0 = time.time()
final_params = train_mlp(X_train_s, y_train, X_val_s, y_val, seed=SEED, h1=128, h2=64, dropout_p=0.3)
train_time_s = time.time() - t0

n_params = sum(p.size for p in final_params.values())

t0 = time.time()
mean_probs, all_probs = mlp_proba(final_params, X_test_s, dropout_p=0.3, mc_passes=100)
mc100_time_s = time.time() - t0
t0 = time.time()
det_probs, _ = mlp_proba(final_params, X_test_s, dropout_p=0.3, mc_passes=0)
det_time_s = time.time() - t0

preds_mc = mean_probs.argmax(1)
preds_det = det_probs.argmax(1)
eps = 1e-12
pred_entropy = -np.sum(mean_probs*np.log(mean_probs+eps), axis=1)
per_pass_entropy = -np.sum(all_probs*np.log(all_probs+eps), axis=2)
aleatoric = per_pass_entropy.mean(axis=0)
epistemic = pred_entropy - aleatoric
confidence = mean_probs.max(1)

print(f"n_params={n_params}  train_time={train_time_s:.2f}s  "
      f"det_inference={det_time_s*1000:.2f}ms  mc100_inference={mc100_time_s*1000:.2f}ms  "
      f"overhead_factor={mc100_time_s/max(det_time_s,1e-9):.1f}x")
print(f"Deterministic acc={accuracy_score(y_test,preds_det):.4f}  MC-mean acc={accuracy_score(y_test,preds_mc):.4f}")

# =============================================================================
# SECTION A: Selective Prediction / Risk-Coverage Analysis
# =============================================================================
order = np.argsort(pred_entropy)  # ascending uncertainty = most confident first
n = len(y_test)
coverages = np.arange(1, n+1) / n
sel_acc, sel_f1, sel_err = [], [], []
for k in range(1, n+1):
    idx = order[:k]
    acc_k = accuracy_score(y_test[idx], preds_mc[idx])
    f1_k = f1_score(y_test[idx], preds_mc[idx], average="macro")
    sel_acc.append(acc_k); sel_f1.append(f1_k); sel_err.append(1-acc_k)

selective_df = pd.DataFrame({"coverage": coverages, "accuracy": sel_acc, "macro_f1": sel_f1, "error_rate": sel_err})
selective_df.to_csv(f"{OUT}/selective_prediction.csv", index=False)

# AURC (area under risk-coverage curve) -- lower is better
aurc = np.trapezoid(sel_err, coverages) if hasattr(np, "trapezoid") else np.trapz(sel_err, coverages)
full_acc = sel_acc[-1]
print(f"AURC={aurc:.4f}  full-coverage accuracy={full_acc:.4f}")

plt.figure(figsize=(7,5.2))
plt.plot(coverages, sel_acc, color="#1F4E79", lw=2, label="Accuracy")
plt.plot(coverages, sel_f1, color="#C55A11", lw=2, linestyle="--", label="Macro F1")
plt.axhline(full_acc, color="grey", linestyle=":", lw=1, label=f"Full-coverage accuracy ({full_acc:.3f})")
plt.xlabel("Coverage (fraction of test samples retained, most-confident first)")
plt.ylabel("Performance")
plt.title(f"Selective Prediction: Risk\u2013Coverage Curve  (AURC={aurc:.4f})")
plt.legend(); plt.grid(alpha=0.25)
plt.tight_layout(); plt.savefig(f"{OUT}/selective_prediction.png", dpi=200); plt.close()

# =============================================================================
# SECTION B: Uncertainty Quality Analysis (low/medium/high groups)
# =============================================================================
terciles = np.quantile(pred_entropy, [1/3, 2/3])
group = np.where(pred_entropy <= terciles[0], "Low", np.where(pred_entropy <= terciles[1], "Medium", "High"))
unc_rows = []
for g in ["Low", "Medium", "High"]:
    m = group == g
    acc_g = accuracy_score(y_test[m], preds_mc[m])
    f1_g = f1_score(y_test[m], preds_mc[m], average="macro") if m.sum() > 0 else np.nan
    err_g = 1 - acc_g
    conf_g = confidence[m].mean()
    # class distribution within group (which classes dominate high-uncertainty group)
    vals, counts = np.unique(y_test[m], return_counts=True)
    class_dist = {str(v): int(c) for v, c in zip(vals, counts)}
    unc_rows.append((g, int(m.sum()), acc_g, f1_g, err_g, conf_g, json.dumps(class_dist)))
unc_df = pd.DataFrame(unc_rows, columns=["Uncertainty_Group","N","Accuracy","Macro_F1","Error_Rate","Mean_Confidence","Class_Distribution"])
unc_df.to_csv(f"{OUT}/uncertainty_groups.csv", index=False)
print("\nUncertainty group analysis:\n", unc_df[["Uncertainty_Group","N","Accuracy","Macro_F1","Error_Rate"]].to_string(index=False))

# error samples: which classes are over-represented among incorrect + high-uncertainty
incorrect_mask = preds_mc != y_test
high_unc_mask = group == "High"
overlap = incorrect_mask & high_unc_mask
print(f"Incorrect predictions: {incorrect_mask.sum()}  High-uncertainty: {high_unc_mask.sum()}  "
      f"Incorrect AND High-uncertainty: {overlap.sum()}  "
      f"(P(high-unc | incorrect) = {overlap.sum()/max(incorrect_mask.sum(),1):.3f})")

plt.figure(figsize=(7,5))
sns.barplot(data=unc_df, x="Uncertainty_Group", y="Accuracy", order=["Low","Medium","High"], color="#1F4E79")
plt.ylim(0,1.02); plt.ylabel("Accuracy"); plt.title("Prediction Accuracy by MC-Dropout Uncertainty Tercile")
plt.tight_layout(); plt.savefig(f"{OUT}/uncertainty_groups.png", dpi=200); plt.close()

# =============================================================================
# SECTION C: MC-Dropout Convergence across T
# =============================================================================
T_values = [10, 25, 50, 100, 200]
conv_rows = []
r_check = np.random.default_rng(SEED)
# use a fixed large pool of passes and take prefixes, for a fair, efficient convergence check
max_T = max(T_values)
_, all_probs_max = mlp_proba(final_params, X_test_s, dropout_p=0.3, mc_passes=max_T, seed=SEED)
for T in T_values:
    probs_T = all_probs_max[:T]
    mean_T = probs_T.mean(axis=0)
    ent_T = -np.sum(mean_T*np.log(mean_T+eps), axis=1)
    acc_T = accuracy_score(y_test, mean_T.argmax(1))
    # stability: std of predictive entropy estimate across 10 bootstrap resamples of the T passes
    boot_ents = []
    for b in range(10):
        bs_idx = r_check.integers(0, T, size=T)
        m_bs = probs_T[bs_idx].mean(axis=0)
        e_bs = -np.sum(m_bs*np.log(m_bs+eps), axis=1)
        boot_ents.append(e_bs.mean())
    conv_rows.append((T, acc_T, ent_T.mean(), np.std(boot_ents)))
conv_df = pd.DataFrame(conv_rows, columns=["T","Accuracy","Mean_Predictive_Entropy","Bootstrap_SD_of_Mean_Entropy"])
conv_df.to_csv(f"{OUT}/mc_convergence.csv", index=False)
print("\nMC-Dropout convergence:\n", conv_df.to_string(index=False))

fig, axes = plt.subplots(1, 2, figsize=(12,4.5))
axes[0].plot(conv_df["T"], conv_df["Accuracy"], marker="o", color="#1F4E79")
axes[0].set_xlabel("Number of MC passes (T)"); axes[0].set_ylabel("Accuracy"); axes[0].set_title("Accuracy vs. T")
axes[1].plot(conv_df["T"], conv_df["Mean_Predictive_Entropy"], marker="o", color="#C55A11", label="Mean entropy")
axes[1].fill_between(conv_df["T"], conv_df["Mean_Predictive_Entropy"]-conv_df["Bootstrap_SD_of_Mean_Entropy"],
                      conv_df["Mean_Predictive_Entropy"]+conv_df["Bootstrap_SD_of_Mean_Entropy"], alpha=0.25, color="#C55A11")
axes[1].set_xlabel("Number of MC passes (T)"); axes[1].set_ylabel("Predictive entropy (nats)"); axes[1].set_title("Uncertainty Stability vs. T")
plt.tight_layout(); plt.savefig(f"{OUT}/mc_convergence.png", dpi=200); plt.close()

# =============================================================================
# SECTION D: Ablation Study
# =============================================================================
ablation_rows = []

def eval_config(params, dropout_p, mc_passes, label):
    t0=time.time()
    mp, ap = mlp_proba(params, X_test_s, dropout_p=dropout_p, mc_passes=mc_passes)
    infer_t = time.time()-t0
    pr = mp.argmax(1)
    acc = accuracy_score(y_test, pr); f1 = f1_score(y_test, pr, average="macro")
    if ap is not None:
        ent = -np.sum(mp*np.log(mp+eps), axis=1)
        pp_ent = -np.sum(ap*np.log(ap+eps), axis=2)
        ale = pp_ent.mean(0); epi = ent-ale
        mean_epi, mean_ale = epi.mean(), ale.mean()
    else:
        mean_epi, mean_ale = np.nan, np.nan
    n_bins=10; bins=np.linspace(0,1,n_bins+1); conf_ = mp.max(1)
    bin_acc,bin_conf,bin_n=[],[],[]
    correct_ = (pr==y_test).astype(float)
    for i in range(n_bins):
        m_ = (conf_>bins[i])&(conf_<=bins[i+1])
        if m_.sum()>0:
            bin_acc.append(correct_[m_].mean()); bin_conf.append(conf_[m_].mean()); bin_n.append(m_.sum())
    tot=len(conf_); ece = sum(c/tot*abs(a-cf) for a,cf,c in zip(bin_acc,bin_conf,bin_n))
    ablation_rows.append((label, dropout_p, mc_passes if mc_passes else 1, acc, f1, mean_epi, mean_ale, ece, infer_t*1000))

# 1: no dropout at all (train with p=0, eval deterministic)
p_nodrop = train_mlp(X_train_s, y_train, X_val_s, y_val, seed=SEED, dropout_p=0.0)
eval_config(p_nodrop, dropout_p=0.0, mc_passes=0, label="No Dropout (baseline MLP)")

# 2: standard dropout, deterministic eval (dropout off at test)
eval_config(final_params, dropout_p=0.3, mc_passes=0, label="Dropout(0.3), standard eval (dropout OFF at test)")

# 3: MC-Dropout (proposed), T=100
eval_config(final_params, dropout_p=0.3, mc_passes=100, label="Dropout(0.3) + MC-Dropout (T=100)")

# 4: different dropout probabilities, all with MC-Dropout T=100
for dp in [0.1, 0.2, 0.5]:
    p_dp = train_mlp(X_train_s, y_train, X_val_s, y_val, seed=SEED, dropout_p=dp)
    eval_config(p_dp, dropout_p=dp, mc_passes=100, label=f"MC-Dropout, p={dp}")

# 5: class-weighted vs unweighted training (with MC-Dropout T=100, p=0.3)
p_weighted = train_mlp(X_train_s, y_train, X_val_s, y_val, seed=SEED, dropout_p=0.3, weighted=True)
eval_config(p_weighted, dropout_p=0.3, mc_passes=100, label="Class-weighted training + MC-Dropout")

ablation_df = pd.DataFrame(ablation_rows, columns=["Configuration","Dropout_p","MC_Passes","Accuracy","Macro_F1",
                                                     "Mean_Epistemic","Mean_Aleatoric","ECE","Inference_Time_ms"])
ablation_df.to_csv(f"{OUT}/ablation_study.csv", index=False)
print("\nAblation study:\n", ablation_df.to_string(index=False))

plt.figure(figsize=(9,5))
sns.barplot(data=ablation_df, y="Configuration", x="Accuracy", color="#1F4E79")
plt.xlim(0.5,1.0); plt.title("Ablation Study: Accuracy by Configuration")
plt.tight_layout(); plt.savefig(f"{OUT}/ablation_study.png", dpi=200); plt.close()

# =============================================================================
# SECTION E: Calibration Comparison Across All Models
# =============================================================================
def calibration_metrics(y_true, probs, n_bins=10):
    preds = probs.argmax(1); conf = probs.max(1)
    correct = (preds==y_true).astype(float)
    bins = np.linspace(0,1,n_bins+1)
    bin_acc,bin_conf,bin_n=[],[],[]
    for i in range(n_bins):
        m = (conf>bins[i])&(conf<=bins[i+1])
        if m.sum()>0:
            bin_acc.append(correct[m].mean()); bin_conf.append(conf[m].mean()); bin_n.append(m.sum())
    tot=len(conf)
    ece = sum(c/tot*abs(a-cf) for a,cf,c in zip(bin_acc,bin_conf,bin_n))
    mce = max(abs(a-cf) for a,cf in zip(bin_acc,bin_conf)) if bin_acc else np.nan
    y_bin = one_hot(y_true, n_classes)
    brier = np.mean(np.sum((probs - y_bin)**2, axis=1))
    nll = log_loss(y_true, probs, labels=list(range(n_classes)))
    return ece, mce, brier, nll

calib_rows = []
calib_rows.append(("MLP (deterministic)", *calibration_metrics(y_test, det_probs)))
calib_rows.append(("MLP + MC-Dropout (T=100)", *calibration_metrics(y_test, mean_probs)))

scaler_full = StandardScaler().fit(X_train)  # baselines use train-only fit, consistent with MLP
Xtr_b, Xte_b = scaler_full.transform(X_train), scaler_full.transform(X_test)
lr_clf = LogisticRegression(max_iter=2000, random_state=SEED).fit(Xtr_b, y_train)
rf_clf = RandomForestClassifier(n_estimators=300, random_state=SEED).fit(Xtr_b, y_train)
svm_clf = SVC(kernel="rbf", probability=True, random_state=SEED).fit(Xtr_b, y_train)
gb_clf = GradientBoostingClassifier(random_state=SEED).fit(Xtr_b, y_train)
for name, clf in [("Logistic Regression", lr_clf), ("Random Forest", rf_clf), ("SVM (RBF)", svm_clf), ("Gradient Boosting", gb_clf)]:
    proba = clf.predict_proba(Xte_b)
    calib_rows.append((name, *calibration_metrics(y_test, proba)))

calib_df = pd.DataFrame(calib_rows, columns=["Model","ECE","MCE","Brier_Score","NLL"])
calib_df.to_csv(f"{OUT}/calibration_comparison.csv", index=False)
print("\nCalibration comparison:\n", calib_df.to_string(index=False))

plt.figure(figsize=(9,5))
x = np.arange(len(calib_df))
width=0.2
plt.bar(x-width, calib_df["ECE"], width, label="ECE", color="#1F4E79")
plt.bar(x, calib_df["MCE"], width, label="MCE", color="#C55A11")
plt.bar(x+width, calib_df["Brier_Score"], width, label="Brier", color="#548235")
plt.xticks(x, calib_df["Model"], rotation=25, ha="right")
plt.ylabel("Error"); plt.title("Calibration Metrics Across Models"); plt.legend()
plt.tight_layout(); plt.savefig(f"{OUT}/calibration_comparison.png", dpi=200); plt.close()

# =============================================================================
# SECTION F: Robustness -- repeated seeds, reduced training data, feature noise
# =============================================================================
# F1: repeated seeds
seed_accs, seed_f1s = [], []
for s in [1,2,3,4,5]:
    p_s = train_mlp(X_train_s, y_train, X_val_s, y_val, seed=s, dropout_p=0.3)
    mp_s,_ = mlp_proba(p_s, X_test_s, dropout_p=0.3, mc_passes=50, seed=s)
    pr_s = mp_s.argmax(1)
    seed_accs.append(accuracy_score(y_test, pr_s)); seed_f1s.append(f1_score(y_test, pr_s, average="macro"))
seed_accs = np.array(seed_accs); seed_f1s = np.array(seed_f1s)
ci95 = 1.96 * seed_accs.std(ddof=1) / np.sqrt(len(seed_accs))
print(f"\nRepeated-seed robustness: acc={seed_accs.mean():.4f}\u00b1{seed_accs.std():.4f}  "
      f"95% CI=[{seed_accs.mean()-ci95:.4f}, {seed_accs.mean()+ci95:.4f}]")

# F2: reduced training data
reduce_rows = []
for frac in [0.25, 0.5, 0.75, 1.0]:
    n_use = int(len(X_train_s)*frac)
    rng_local = np.random.default_rng(SEED)
    idx = rng_local.choice(len(X_train_s), size=n_use, replace=False)
    p_r = train_mlp(X_train_s[idx], y_train[idx], X_val_s, y_val, seed=SEED, dropout_p=0.3)
    mp_r,_ = mlp_proba(p_r, X_test_s, dropout_p=0.3, mc_passes=50)
    pr_r = mp_r.argmax(1)
    ent_r = -np.sum(mp_r*np.log(mp_r+eps), axis=1)
    reduce_rows.append((frac, n_use, accuracy_score(y_test, pr_r), f1_score(y_test, pr_r, average="macro"), ent_r.mean()))
reduce_df = pd.DataFrame(reduce_rows, columns=["Train_Fraction","N_Train","Accuracy","Macro_F1","Mean_Predictive_Entropy"])
reduce_df.to_csv(f"{OUT}/robustness_reduced_data.csv", index=False)
print("\nReduced training data:\n", reduce_df.to_string(index=False))

# F3: feature noise perturbation (Gaussian noise added to standardized test features)
noise_rows = []
rng_noise = np.random.default_rng(SEED)
for sigma in [0.0, 0.25, 0.5, 1.0, 2.0]:
    X_noisy = X_test_s + rng_noise.normal(0, sigma, X_test_s.shape)
    mp_n,_ = mlp_proba(final_params, X_noisy, dropout_p=0.3, mc_passes=50)
    pr_n = mp_n.argmax(1)
    ent_n = -np.sum(mp_n*np.log(mp_n+eps), axis=1)
    noise_rows.append((sigma, accuracy_score(y_test, pr_n), f1_score(y_test, pr_n, average="macro"), ent_n.mean()))
noise_df = pd.DataFrame(noise_rows, columns=["Noise_Sigma","Accuracy","Macro_F1","Mean_Predictive_Entropy"])
noise_df.to_csv(f"{OUT}/robustness_noise.csv", index=False)
print("\nFeature-noise robustness:\n", noise_df.to_string(index=False))

fig, axes = plt.subplots(1, 2, figsize=(12,4.5))
axes[0].plot(reduce_df["Train_Fraction"], reduce_df["Accuracy"], marker="o", color="#1F4E79")
axes[0].set_xlabel("Fraction of training data used"); axes[0].set_ylabel("Test accuracy"); axes[0].set_title("Robustness to Reduced Training Data")
ax2 = axes[1]
ax2.plot(noise_df["Noise_Sigma"], noise_df["Accuracy"], marker="o", color="#1F4E79", label="Accuracy")
ax2b = ax2.twinx()
ax2b.plot(noise_df["Noise_Sigma"], noise_df["Mean_Predictive_Entropy"], marker="s", color="#C55A11", label="Predictive entropy")
ax2.set_xlabel("Gaussian noise \u03c3 (standardized feature units)"); ax2.set_ylabel("Accuracy", color="#1F4E79")
ax2b.set_ylabel("Mean predictive entropy", color="#C55A11")
ax2.set_title("Accuracy & Uncertainty under Feature Noise")
plt.tight_layout(); plt.savefig(f"{OUT}/robustness_combined.png", dpi=200); plt.close()

# =============================================================================
# SECTION G: Class-wise metrics (precision/recall/F1/sensitivity/specificity) + PR curves
# =============================================================================
cm = confusion_matrix(y_test, preds_mc, labels=list(range(n_classes)))
class_rows = []
y_test_bin = label_binarize(y_test, classes=list(range(n_classes)))
for c in range(n_classes):
    tp = cm[c,c]; fn = cm[c,:].sum()-tp; fp = cm[:,c].sum()-tp; tn = cm.sum()-tp-fn-fp
    prec = tp/(tp+fp) if (tp+fp)>0 else np.nan
    rec = tp/(tp+fn) if (tp+fn)>0 else np.nan   # sensitivity
    spec = tn/(tn+fp) if (tn+fp)>0 else np.nan
    f1c = 2*prec*rec/(prec+rec) if (prec and rec and (prec+rec)>0) else np.nan
    auc_c = roc_auc_score(y_test_bin[:,c], mean_probs[:,c])
    ap_c = average_precision_score(y_test_bin[:,c], mean_probs[:,c])
    class_rows.append((c, int(cm[c,:].sum()), prec, rec, spec, f1c, auc_c, ap_c))
classwise_df = pd.DataFrame(class_rows, columns=["Class","N","Precision","Recall_Sensitivity","Specificity","F1","ROC_AUC","PR_AUC"])
classwise_df.to_csv(f"{OUT}/classwise_metrics.csv", index=False)
print("\nClass-wise metrics:\n", classwise_df.to_string(index=False))

plt.figure(figsize=(7.5,6))
colors = sns.color_palette("husl", n_classes)
for c in range(n_classes):
    prec_c, rec_c, _ = precision_recall_curve(y_test_bin[:,c], mean_probs[:,c])
    plt.plot(rec_c, prec_c, color=colors[c], lw=2, label=f"Class {c} (AP={classwise_df.loc[c,'PR_AUC']:.3f})")
plt.xlabel("Recall"); plt.ylabel("Precision"); plt.title("Precision\u2013Recall Curves (One-vs-Rest)")
plt.legend(fontsize=9, loc="lower left")
plt.tight_layout(); plt.savefig(f"{OUT}/pr_curves.png", dpi=200); plt.close()

# =============================================================================
# SECTION H: Overall metrics extended (MCC, Cohen's kappa, balanced accuracy, PR-AUC macro)
# =============================================================================
mcc = matthews_corrcoef(y_test, preds_mc)
kappa = cohen_kappa_score(y_test, preds_mc)
bal_acc = balanced_accuracy_score(y_test, preds_mc)
macro_pr_auc = classwise_df["PR_AUC"].mean()
overall_extended = dict(mcc=mcc, cohen_kappa=kappa, balanced_accuracy=bal_acc, macro_pr_auc=macro_pr_auc,
                         aurc=aurc, n_params=int(n_params), train_time_s=train_time_s,
                         det_inference_ms=det_time_s*1000, mc100_inference_ms=mc100_time_s*1000,
                         mc_overhead_factor=mc100_time_s/max(det_time_s,1e-9),
                         repeated_seed_acc_mean=float(seed_accs.mean()), repeated_seed_acc_std=float(seed_accs.std()),
                         repeated_seed_acc_ci95_low=float(seed_accs.mean()-ci95), repeated_seed_acc_ci95_high=float(seed_accs.mean()+ci95),
                         p_high_unc_given_incorrect=float(overlap.sum()/max(incorrect_mask.sum(),1)))
with open(f"{OUT}/overall_extended_metrics.json","w") as f:
    json.dump(overall_extended, f, indent=2)
print("\nOverall extended metrics:\n", json.dumps(overall_extended, indent=2))

print(f"\nAll Section A-H artifacts saved to {OUT}/")
print("\nNOTE: XGBoost, LightGBM, and SHAP are not installed and no internet access is available in "
      "this environment to install them; these were therefore not run and are not reported. No second "
      "compatible genetic-disease dataset was available for cross-dataset external validation.")

# =============================================================================
# SECTION I: Regenerate ALL core figures/metrics from THIS corrected 3-hidden-layer
# (128/64/32) model so every number and figure in the manuscript traces to a single,
# architecturally-consistent source (earlier drafts inconsistently used a 2-hidden-layer
# 64/32 network for final evaluation despite documenting 128/64/32 -- corrected here).
# =============================================================================
print("\n=== Regenerating core manuscript figures from the corrected, consistent model ===")

# retrain with history for the training-curve figure
def train_mlp_with_history(X_train, y_train, X_val, y_val, seed, h1=128, h2=64, h3=32,
                            dropout_p=0.3, epochs=300, patience=25, lr=1e-3, batch_size=32):
    params = init_params(X_train.shape[1], h1, h2, h3, seed)
    r = np.random.default_rng(seed)
    m = {k: np.zeros_like(v) for k,v in params.items()}
    v = {k: np.zeros_like(v) for k,v in params.items()}
    beta1,beta2,eps_adam = 0.9,0.999,1e-8
    t = 0
    Y_train = one_hot(y_train, n_classes)
    best_val, best_params, no_improve = np.inf, None, 0
    N = X_train.shape[0]
    hist = {"train_loss":[], "val_loss":[], "train_acc":[], "val_acc":[]}
    for epoch in range(epochs):
        idx = r.permutation(N)
        Xs, Ys = X_train[idx], Y_train[idx]
        losses=[]
        for start in range(0, N, batch_size):
            xb, yb = Xs[start:start+batch_size], Ys[start:start+batch_size]
            probs, cache = forward(xb, params, True, r, dropout_p)
            losses.append(cross_entropy(probs, yb))
            grads = backward(yb, cache, params)
            t += 1
            for k in params:
                m[k] = beta1*m[k]+(1-beta1)*grads[k]
                v[k] = beta2*v[k]+(1-beta2)*(grads[k]**2)
                mhat = m[k]/(1-beta1**t); vhat = v[k]/(1-beta2**t)
                params[k] -= lr*mhat/(np.sqrt(vhat)+eps_adam)
        train_probs,_ = forward(X_train, params, False, r)
        val_probs,_ = forward(X_val, params, False, r)
        val_loss = cross_entropy(val_probs, one_hot(y_val, n_classes))
        hist["train_loss"].append(np.mean(losses)); hist["val_loss"].append(val_loss)
        hist["train_acc"].append(accuracy_score(y_train, train_probs.argmax(1)))
        hist["val_acc"].append(accuracy_score(y_val, val_probs.argmax(1)))
        if val_loss < best_val - 1e-4:
            best_val, best_params, no_improve = val_loss, {k: v_.copy() for k,v_ in params.items()}, 0
        else:
            no_improve += 1
            if no_improve >= patience:
                break
    return best_params, hist

_, hist = train_mlp_with_history(X_train_s, y_train, X_val_s, y_val, seed=SEED)

fig, axes = plt.subplots(1, 2, figsize=(12, 4))
axes[0].plot(hist["train_loss"], label="train"); axes[0].plot(hist["val_loss"], label="val")
axes[0].set_title("Loss"); axes[0].set_xlabel("Epoch"); axes[0].legend()
axes[1].plot(hist["train_acc"], label="train"); axes[1].plot(hist["val_acc"], label="val")
axes[1].set_title("Accuracy"); axes[1].set_xlabel("Epoch"); axes[1].legend()
plt.tight_layout(); plt.savefig(f"{OUT}/training_curves.png", dpi=150); plt.close()

cm_final = confusion_matrix(y_test, preds_mc, labels=list(range(n_classes)))
plt.figure(figsize=(6,5))
sns.heatmap(cm_final, annot=True, fmt="d", cmap="Blues")
plt.title("Confusion Matrix (Final Held-out Test Set)")
plt.xlabel("Predicted"); plt.ylabel("True")
plt.tight_layout(); plt.savefig(f"{OUT}/confusion_matrix.png", dpi=150); plt.close()

correct_mask_final = preds_mc == y_test
plt.figure(figsize=(7,5))
sns.histplot(pred_entropy[correct_mask_final], color="green", label="Correct", stat="density", kde=True, alpha=0.5)
sns.histplot(pred_entropy[~correct_mask_final], color="red", label="Incorrect", stat="density", kde=True, alpha=0.5)
plt.xlabel("Predictive entropy"); plt.title("Uncertainty: correct vs incorrect predictions"); plt.legend()
plt.tight_layout(); plt.savefig(f"{OUT}/uncertainty_correct_vs_incorrect.png", dpi=150); plt.close()

plt.figure(figsize=(6,5))
sc = plt.scatter(confidence, pred_entropy, c=epistemic, cmap="viridis", s=15)
plt.colorbar(sc, label="Epistemic uncertainty")
plt.xlabel("Max predicted probability (confidence)"); plt.ylabel("Predictive entropy")
plt.title("Confidence vs Uncertainty")
plt.tight_layout(); plt.savefig(f"{OUT}/confidence_vs_uncertainty.png", dpi=150); plt.close()

n_bins=10
bins = np.linspace(0,1,n_bins+1)
bin_acc, bin_conf, bin_count = [], [], []
accs_bin = correct_mask_final.astype(float)
for i in range(n_bins):
    m = (confidence>bins[i]) & (confidence<=bins[i+1])
    if m.sum()>0:
        bin_acc.append(accs_bin[m].mean()); bin_conf.append(confidence[m].mean()); bin_count.append(m.sum())
    else:
        bin_acc.append(np.nan); bin_conf.append(np.nan); bin_count.append(0)
total_n=len(confidence)
ece_final = sum(c/total_n*abs(a-cf) for a,cf,c in zip(bin_acc,bin_conf,bin_count) if not np.isnan(a))
plt.figure(figsize=(6,5))
plt.plot([0,1],[0,1],"k--", label="Perfect calibration")
plt.plot(bin_conf, bin_acc, marker="o", label=f"Model (ECE={ece_final:.3f})")
plt.xlabel("Mean predicted confidence"); plt.ylabel("Empirical accuracy"); plt.title("Reliability Diagram"); plt.legend()
plt.tight_layout(); plt.savefig(f"{OUT}/calibration_curve.png", dpi=150); plt.close()

# ROC curves
plt.figure(figsize=(7,6))
colors = sns.color_palette("husl", n_classes)
from sklearn.metrics import roc_curve, auc as auc_fn
for i in range(n_classes):
    fpr, tpr, _ = roc_curve(y_test_bin[:, i], mean_probs[:, i])
    roc_auc_i = auc_fn(fpr, tpr)
    plt.plot(fpr, tpr, color=colors[i], lw=2, label=f"Class {i} (AUC={roc_auc_i:.3f})")
plt.plot([0,1],[0,1],"k--", lw=1)
plt.xlabel("False Positive Rate"); plt.ylabel("True Positive Rate")
plt.title("Multi-class ROC Curves (One-vs-Rest)")
plt.legend(loc="lower right", fontsize=9)
plt.tight_layout(); plt.savefig(f"{OUT}/roc_curves.png", dpi=150); plt.close()
per_class_auc_final = [roc_auc_score(y_test_bin[:,c], mean_probs[:,c]) for c in range(n_classes)]

# 5-fold CV + baselines + McNemar, rerun with the CORRECT 3-hidden-layer architecture
skf = StratifiedKFold(n_splits=5, shuffle=True, random_state=SEED)
cv_models = {"MLP (proposed)": [], "Logistic Regression": [], "Random Forest": [], "SVM (RBF)": [], "Gradient Boosting": []}
cv_f1 = {k: [] for k in cv_models}
mlp_fold_preds, mlp_fold_true = [], []
base_fold_preds = {k: [] for k in cv_models if k != "MLP (proposed)"}
fold_i = 0
for tr_idx, te_idx in skf.split(X_all, y_all):
    fold_i += 1
    Xtr_raw, Xte_raw = X_all[tr_idx], X_all[te_idx]
    ytr, yte = y_all[tr_idx], y_all[te_idx]
    Xtr2, Xval2, ytr2, yval2 = train_test_split(Xtr_raw, ytr, test_size=0.15, random_state=SEED, stratify=ytr)
    sc = StandardScaler().fit(Xtr2)
    Xtr2s, Xval2s = sc.transform(Xtr2), sc.transform(Xval2)
    p_fold = train_mlp(Xtr2s, ytr2, Xval2s, yval2, seed=SEED+fold_i, h1=128, h2=64, h3=32, dropout_p=0.3)
    mp_fold,_ = mlp_proba(p_fold, sc.transform(Xte_raw), dropout_p=0.3, mc_passes=0)
    pr_fold = mp_fold.argmax(1)
    cv_models["MLP (proposed)"].append(accuracy_score(yte, pr_fold))
    cv_f1["MLP (proposed)"].append(f1_score(yte, pr_fold, average="macro"))
    mlp_fold_preds.extend(pr_fold); mlp_fold_true.extend(yte)

    sc_full = StandardScaler().fit(Xtr_raw)
    Xtr_full_s, Xte_full_s = sc_full.transform(Xtr_raw), sc_full.transform(Xte_raw)
    lr_c = LogisticRegression(max_iter=2000, random_state=SEED).fit(Xtr_full_s, ytr)
    rf_c = RandomForestClassifier(n_estimators=300, random_state=SEED).fit(Xtr_full_s, ytr)
    svm_c = SVC(kernel="rbf", probability=True, random_state=SEED).fit(Xtr_full_s, ytr)
    gb_c = GradientBoostingClassifier(random_state=SEED).fit(Xtr_full_s, ytr)
    for name, clf in [("Logistic Regression", lr_c), ("Random Forest", rf_c), ("SVM (RBF)", svm_c), ("Gradient Boosting", gb_c)]:
        pp = clf.predict(Xte_full_s)
        cv_models[name].append(accuracy_score(yte, pp)); cv_f1[name].append(f1_score(yte, pp, average="macro"))
        base_fold_preds[name].extend(pp)
    print(f"CV fold {fold_i}: " + ", ".join(f"{k}={cv_models[k][-1]:.3f}" for k in cv_models))

cv_summary = pd.DataFrame([(k, np.mean(v), np.std(v), np.mean(cv_f1[k]), np.std(cv_f1[k])) for k,v in cv_models.items()],
                           columns=["Model","Acc_mean","Acc_std","F1_mean","F1_std"])
cv_summary.to_csv(f"{OUT}/cv_model_comparison.csv", index=False)
print("\nCorrected CV summary:\n", cv_summary.to_string(index=False))

from scipy.stats import chi2, binomtest
def mcnemar_test(n01, n10, thr=25):
    n=n01+n10
    if n==0: return 0.0,1.0
    if n<thr:
        return float(min(n01,n10)), binomtest(min(n01,n10), n, 0.5, alternative="two-sided").pvalue
    stat=(abs(n01-n10)-1)**2/n
    return stat, 1-chi2.cdf(stat, df=1)

mlp_arr = np.array(mlp_fold_preds); true_arr = np.array(mlp_fold_true)
mcnemar_rows=[]
for name in base_fold_preds:
    base_arr = np.array(base_fold_preds[name])
    mc_ = mlp_arr==true_arr; bc_ = base_arr==true_arr
    n01=int(np.sum(mc_ & ~bc_)); n10=int(np.sum(~mc_ & bc_))
    stat,p = mcnemar_test(n01,n10)
    mcnemar_rows.append((name, stat, p, n01, n10))
    print(f"McNemar MLP vs {name}: stat={stat:.3f} p={p:.4f} (n01={n01}, n10={n10})")
mcnemar_df2 = pd.DataFrame(mcnemar_rows, columns=["Comparison","Statistic","p_value","MLP_only_correct","Baseline_only_correct"])
mcnemar_df2.to_csv(f"{OUT}/mcnemar_tests.csv", index=False)

# feature importance (unchanged methodology, rerun for completeness/consistency)
rf_full = RandomForestClassifier(n_estimators=300, random_state=SEED).fit(X_train_s, y_train)
from sklearn.inspection import permutation_importance
perm = permutation_importance(rf_full, X_test_s, y_test, n_repeats=30, random_state=SEED, scoring="f1_macro")
importances = pd.Series(perm.importances_mean, index=feature_cols).sort_values(ascending=True)
plt.figure(figsize=(7,6))
importances.plot(kind="barh", color="#1F4E79")
plt.xlabel("Permutation importance (\u0394 macro-F1)")
plt.title("Feature Importance (Random Forest, permutation-based)")
plt.tight_layout(); plt.savefig(f"{OUT}/feature_importance.png", dpi=150); plt.close()
importances.sort_values(ascending=False).to_csv(f"{OUT}/feature_importance.csv")

plt.figure(figsize=(8,5))
names = list(cv_models.keys())
means = [np.mean(cv_models[n]) for n in names]; stds=[np.std(cv_models[n]) for n in names]
colors_bar = ["#1F4E79" if n=="MLP (proposed)" else "#A6A6A6" for n in names]
plt.bar(names, means, yerr=stds, capsize=5, color=colors_bar)
plt.ylabel("5-fold CV Accuracy"); plt.xticks(rotation=20, ha="right")
plt.title("Model Comparison: 5-fold Stratified Cross-Validation"); plt.ylim(0.5,1.0)
plt.tight_layout(); plt.savefig(f"{OUT}/model_comparison_bar.png", dpi=150); plt.close()

# consolidated, corrected final metrics
final_metrics_corrected = dict(
    accuracy_deterministic=float(accuracy_score(y_test, preds_det)),
    accuracy_mc_mean=float(accuracy_score(y_test, preds_mc)),
    f1_macro=float(f1_score(y_test, preds_mc, average="macro")),
    precision_macro=float(precision_score(y_test, preds_mc, average="macro")),
    recall_macro=float(recall_score(y_test, preds_mc, average="macro")),
    macro_auc=float(roc_auc_score(y_test_bin, mean_probs, multi_class="ovr", average="macro")),
    per_class_auc={class_names[c]: per_class_auc_final[c] for c in range(n_classes)},
    mean_predictive_entropy=float(pred_entropy.mean()),
    mean_epistemic=float(epistemic.mean()),
    mean_aleatoric=float(aleatoric.mean()),
    ece=float(ece_final),
    mcc=float(mcc), cohen_kappa=float(kappa), balanced_accuracy=float(bal_acc), macro_pr_auc=float(macro_pr_auc),
    n_params=int(n_params),
)
with open(f"{OUT}/final_metrics.json","w") as f:
    json.dump(final_metrics_corrected, f, indent=2)
print("\nCORRECTED final metrics (single source of truth for the manuscript):\n", json.dumps(final_metrics_corrected, indent=2))
```
